# Network meta-analysis of randomized controlled trials evaluating long-term outcomes of interventional approaches for great saphenous vein insufficiency

**DOI:** 10.1016/j.jvsv.2026.102553

**Published:** 2026-06-15

**Authors:** Fan Maitri Aldian, Yan Efrata Sembiring, Visuddho Visuddho, Bendix Samarta Witarto, Jason Oktavian Hartanto, Andro Pramana Witarto, Jeffrey Jeswant Dillon

**Affiliations:** a Faculty of Medicine, Universitas Airlangga, Surabaya, Indonesia; b Department of Thoracic Cardiac and Vascular Surgery, Faculty of Medicine, Universitas Airlangga, Surabaya, Indonesia; c Department of Thoracic Cardiac and Vascular Surgery, Dr Soetomo General Academic Hospital, Surabaya, Indonesia; d Department of Internal Medicine, Faculty of Medicine, Universitas Airlangga, Surabaya, Indonesia; e Department of Internal Medicine, Dr Soetomo General Academic Hospital, Surabaya, Indonesia; f Department of Cardiology and Department of Cardiothoracic Surgery, Institut Jantung Negara, Kuala Lumpur, Malaysia

**Keywords:** Great saphenous vein, Guideline, Network meta-analysis, Surgery, Varicose vein

## Abstract

**Objective:**

Great saphenous vein (GSV) insufficiency is a major contributor to chronic venous disease, associated with substantial morbidity and reduced health status. Although various surgical and minimally invasive interventions are available, their long-term efficacy, safety, and recovery profiles remain uncertain. Therefore, this study aims to provide comprehensive evidence to guide clinical decision-making and optimize interventional strategies for GSV insufficiency.

**Methods:**

This network meta-analysis (NMA) was performed following the Preferred Reporting Items for Systematic Reviews and Meta-Analyses-NMA checklist of items. Comprehensive searches were conducted on PubMed, Scopus, Cochrane Central Register of Controlled Trials, ProQuest, and Web of Science from inception to July 11, 2025. Data analyses were performed using RStudio version 4.5.1 (Posit Software). Kaplan-Meier (KM) and log-rank test were applied to evaluate freedom from failure, with individual patient data reconstructed from published KM curves. Clinical outcomes were synthesized using a Frequentist NMA with DerSimonian-Laird random-effects model. Complications were analyzed using meta-proportions with a generalized linear mixed model at the latest follow-up.

**Results:**

A total of 7913 cases of GSV insufficiency from 47 reports from 33 randomized controlled trials were included. KM and log-rank analyses revealed that high ligation and stripping (HL&S) had a significantly higher long-term freedom from failure compared with cyanoacrylate closure [hazard ratio (HR), 1.98; 95% confidence interval (CI), 1.24-3.16; *P* < .001], endovenous laser ablation (EVLA)-1470 nm (HR, 2.37; 95% CI, 1.38-4.06; *P* < .001), and radiofrequency ablation (HR, 2.64; 95% CI, 1.69-4.12; *P* = .002) among patients with Clinical-Etiology-Anatomy-Pathophysiology C2-C5 Classification. Regarding clinical outcomes, electrocoagulation (mean difference, −0.59; 95% CI, −1.11 to −0.06; *P* = .03) showed lowest Venous Clinical Severity Score at 6 months among interventions. Minimally invasive interventions (EVLA-1470, *P* < .001; EVLA-810, *P* = .02; electrocoagulation, *P* = .006) demonstrated a faster return to normal activities compared with HL&S. In terms of complications, HL&S showed higher proportion of ecchymosis (76.37%) and infection (2.35%), whereas ablation procedures demonstrated higher proportion of skin pigmentation (4.34%-11.41%) across interventions.

**Conclusions:**

In individuals with Clinical-Etiology-Anatomy-Pathophysiology stages C2 to C5 disease, HL&S offer higher long-term freedom from failure and Venous Clinical Severity Score improvement, whereas minimally invasive interventions offer faster short-term recovery. These findings provide evidence to ensuring recommendations address both long-term efficacy and patient-centered short-term outcomes.

**Article Highlights**

• **Type of Research**: Network meta-analysis with time-to-event analysis
• **Key Findings**: High ligation and stripping proved to have significantly higher long-term freedom from treatment failure compared with cyanoacrylate closure [hazard ratio (HR), 1.98; 95% confidence interval (CI), 1.24-3.16; *P* < .001], endovenous laser ablation-1470 nm (HR, 2.37; 95% CI, 1.38-4.06; *P* < .001), and radiofrequency ablation (HR, 2.64; 95% CI, 1.69-4.12; *P* = .002).
• **Take Home Message**: Among patients presenting with Clinical-Etiology-Anatomy-Pathophysiology stages C2 to C5, high ligation and stripping provides the greatest benefit in terms of long-term freedom from treatment failure and Venous Clinical Severity Score improvement, whereas minimally invasive interventions offer faster short-term recovery.

Chronic venous disease (CVD), particularly involving the great saphenous vein (GSV), is a widespread and complex vascular condition that exerts a substantial health and socioeconomic burden worldwide.^1^^,^^2^ Epidemiological evidence shows that chronic venous disease affects approximately 18% to 61% of the adult population, influenced by region, age, gender, and lifestyle factors, such as obesity and prolonged standing.^3^^,^^4^ The disease spectrum ranges from mild cosmetic-limited varicosities to debilitating chronic venous insufficiency, including leg edema, skin changes, and venous ulcers, which also markedly impairing patients' overall health status and increasing health care costs significantly.^2^^,^^4^

Despite its high prevalence, uncertainty persists regarding the long-term durability and comparative effectiveness of available interventions for GSV insufficiency. Pharmacological treatments, such as venoactive drugs, may provide symptoms relief and also improve quality-of-life; however, they are lacking in the ability to modify or prevent disease progression.^5^ Meanwhile, a variety of nonpharmacological and interventional treatment options have evolved with varying levels of evidence, including endovenous laser ablation (EVLA), high ligation and stripping (HL&S), ultrasound-guided foam sclerotherapy (UGFS), mechanochemical ablation (MOCA), and newer techniques such as cyanoacrylate closure (CAC).^6^^,^^7^ Current clinical practice guideline from the Society for Vascular Surgery, American Venous Forum, and American Vein and Lymphatic Society, as well as European Society of Vascular Surgery recommend endovenous ablation over other interventional modalities for GSV incompetence, with recommendations supported by moderate-level evidence that recognize limited efficacy and safety profiles and the presence of conflicting supporting evidence.^8^^,^^9^ Reasons for this gap include the technological novelty of emerging treatments, heterogeneity in study designs, lack of robust long-term outcomes, and the complexity of direct comparative trials.^7^^,^^10^ Although compression therapy may provide symptomatic relief and may be used in patients who are not candidates for intervention; however, definitive management is generally achieved through endovenous ablation or surgical intervention.^11^ This study, therefore, undertakes a comprehensive long-term evaluation of the efficacy and safety of a broad array of interventions for GSV incompetence, aiming to fill those critical evidence gaps.

## Methods

### Study design

This study was performed following the Preferred Reporting Items for Systematic Reviews and Meta-Analyses extension for network meta-analysis (NMA)^12^ (Supplementary Table I, online only). The study protocol has been registered on the International Prospective Register of Systematic Reviews (PROSPERO CRD420251123386).

### Search strategies

Literature searches were conducted on PubMed, Scopus, ProQuest, Web of Science, and Cochrane Central Register of Controlled Trials (CENTRAL) from inception to July 11, 2025. Main keywords include “varicose vein,” different types of interventions (eg, “ablation,” “sclerotherapy,” “stripping,” and “ligation”), and “randomized controlled trial.” Medical Subject Headings terms and synonyms combined with Boolean operators were used to construct a specific search term for each database (Supplementary Table II, online only). No language restrictions were set during searches. We additionally searched the reference list of systematic reviews/meta-analyses/eligible studies identified from databases for potential studies.

### Study selection process

Search results from databases were merged collectively using Google Sheets (Google LLC). After duplicates were removed, and the remaining records were screened according to their title and abstract, followed by full-text screening based on the predetermined eligibility criteria. The exclusion reasonings from each screening step were recorded in the spreadsheet and presented in the Preferred Reporting Items for Systematic Reviews and Meta-Analyses flow diagram. The study selection process was conducted independently by five investigators (F.M.A., V.V., B.S.W., A.P.W., and J.O.H.). Discrepancies were resolved through group discussion.

### Eligibility criteria

Eligibility criteria were designed by referring to the Population, Intervention, Comparison, Outcome, and Study Design framework (Table I).^13^ We included studies published in any language that: (1) involved adults aged ≥18 years with GSV insufficiency; (2) investigated different treatment modalities for GSV insufficiency [eg, EVLA, radiofrequency ablation (RFA), MOCA, CAC, UGFS, or HL&S]; (3) reported outcomes on time to treatment failure, Venous Clinical Severity Score (VCSS), time to return to activities, and safety (defined as any complications that occur after treatments); and (4) used a randomized controlled design. Studies with the following characteristics were excluded: (1) irretrievable full-text; (2) review article, case report, case series, conference abstract, or letter to editor; or (3) involved patients with GSV insufficiency clinical class C1 (ie, telangiectasias or reticular veins) and C6 (ie, active venous ulcer) based on the Clinical-Etiology-Anatomy-Pathophysiology (CEAP) classification system. C1 class is not yet considered as a disease entity of varicose veins. In addition, although venous ulcers and varicose veins share the same initial pathophysiologic process, the treatment of active venous ulcers (C6) is often more complex compared with varicose veins, making the management and prognosis differ substantially from varicose vein.^14^^,^^15^

**Table I:** *PICOS* framework

Components of PICOS	Definition
Population	Patients with GSV insufficiency grade C2 to C5
Intervention	EVLA 810 nm, EVLA 1470 nm, EVLA 980 nm, RFA, MOCA, CAC, UGFS, electrocoagulation
Comparison	High ligation and stripping (HL&S)
Outcome	Time to treatment failure, VCSS at 6 months, time to return to activities, and complications
Study design	RCT

*CAC*, Cyanoacrylate closure; *EVLA*, endovenous laser ablation; *GSV*, great saphenous vein; *MOCA*, mechanochemical ablation; *RCT*, randomized controlled trial; *RFA*, radiofrequency ablation; *UGFS*, ultrasound-guided foam sclerotherapy; *VCSS*, the Venous Clinical Severity Score.

### Data extraction process

A prespecified checklist was designed and tabulated within the spreadsheet by FMA. Data extraction was then performed by five investigators (FMA, VV, BSW, APW, and JOH). Eligibility of the data was subsequently checked, and disagreements were immediately resolved. The extracted data included the trial name (or the name of the first author and year of publication if trial name was unavailable), trial protocol identifier, the study eligibility criteria, treatment modalities (including methods, devices, and characteristics), study location, baseline population characteristics (including number of patients, age, and proportion of female, number of right or left treated legs, diameter and length of treated veins, right CEAP clinical class, Aberdeen Varicose Vein Symptom Severity Score, VCSS, Aberdeen Varicose Vein Questionnaire, Euro-quality-of-life-5 dimension score), adherence rate (defined as proportion of participants completed the whole study compared with baseline), type of analysis (intention-to-treat or per-protocol analysis), follow-up assessment tools, time points, and all outcome data required for meta-analysis. Data were then presented in a tabular format for qualitative synthesis.

### Study quality assessment

Assessment of study quality was conducted using the Risk of Bias 2 (RoB-2) tool,^16^ performed by five reviewers (FMA, VV, BSW, APW, and JOH) independently. Any disagreements in judgments were reconciled through a group discussion. The RoB-2 is a revised tool specifically designed to evaluate the RoB of randomized trials arising from the following five domains: (1) randomization process, (2) deviations from intended interventions, (3) missing outcome data, (4) measurement of the outcome, and (5) selection of the reported result. Each domain is assessed using an algorithm with a set of signaling questions, guiding the judgments into different categories: “low risk,” “high risk,” or “some concerns.” Each study was then judged for its overall RoB based on the evaluation in each domain. A study is considered having an overall low RoB if all domains are rated with “low risk.” Studies were judged as having some concerns if at least one domain was rated with “some concerns.” Finally, studies are at a high RoB if at least one domain shows a high RoB or if there were some concerns in multiple domains that may significantly lower the confidence of the study results. The assessment results were then visualized using a weighted summary bar plot and a traffic light plot generated using the “robvis” package in R version 4.5.1 (R Foundation for Statistical Computing).

### Outcomes

The primary outcome of this study was time-to-treatment failure, defined as the presence of venous flow on duplex ultrasound assessment. Complete anatomic occlusion was considered a successful outcome, whereas partial occlusion, neovascularization, partial recanalization, and complete recanalization were classified as treatment failure. Data on time-to-treatment failure were extracted at all available follow-up time points reported in each study. When available, short-, mid-, and long-term follow-up data were included.

Secondary outcomes focused on patient-centered clinical outcomes, including the VCSS at 6 months postintervention and time to return to normal activities. For these outcomes, the analysis was based on the most frequently reported follow-up time point across studies. Postoperative complications were also analyzed, including bleeding, bruising, burn, deep vein thrombosis, dysesthesia, ecchymosis, edema, erythema, hematoma, induration, infection, pain, paresthesia, phlebitis, skin pigmentation, and thrombophlebitis. For this outcome, data were extracted at the latest available follow-up time point reported in each study.

### Statistical analysis

All statistical analyses were performed using RStudio version 4.5.1 (Posit; www.rstudio.com).^17^ For time-to-treatment failure outcome, the Kaplan-Meier (KM) approach was used, as it is considered the gold standard for reporting meta-analyses of time-to-event data.^18^^,^^19^ The construction of KM curves requires individual patient data (IPD) from all included studies. Therefore, IPD data were generated in a three-stage process. The approaches applied for reconstructing IPD in this study have been validated in previous research.^20^ This approach estimates individual event and censoring times by digitizing published KM curves and using the reported numbers at risk over time.

This reconstruction method assumes that censoring occurs uniformly within each time interval that the digitized survival curves accurately reflect the original data, and that censoring is noninformative. These assumptions are well established and widely accepted in secondary survival analyses, and the validity of this approach has been demonstrated in previous methodological studies.^20^ For the randomized controlled trials (RCTs) included in this meta-analysis, these assumptions are considered appropriate, as most studies reported detailed numbers at risk, exhibited low rates of loss to follow-up, and involved stable patient populations undergoing elective interventions.

First, the “dplyr” package (version 1.1.4; Posit Software) was used to derive IPD from the time to treatment failure data reported in each included study.^21^ In this first stage, raw data coordinates (such as time and freedom from failure) were extracted from each intervention arm of the KM curves. These coordinates were then combined with the reported numbers at risk at specific time points to reconstruct IPD. These data were subsequently converted into proportions using the meta-proportion function from the “meta” package (version 8.2-0; R Foundation for Statistical Computing).^22^ Second, proportion data from all-time points for each intervention were analyzed using the “survival” package (version 3.8-3; Mayo Clinic) to construct KM curves and conduct log-rank tests.^23^ Pairwise log-rank tests were also performed to obtain *P* values for each pairwise intervention comparison, with multiple testing adjustments applied using the Holm-Bonferroni method.^24^^,^^25^ Hazard ratio (HR) with Holm-adjusted 95% confidence interval (CI) for the difference between each arms to HL&S were calculated using Cox-proportional hazards model using “coxph” package (version 4.0.2; Terry M. Therneau, Mayo Clinic).^26^ The proportional hazards assumption was evaluated with the Grambsch-Therneau test and diagnostic plots based on Schoenfeld residuals.^27^^,^^28^ In accordance with our protocol, flexible parametric survival models with B-splines and landmark analyses were prespecified as alternatives in cases where the proportional hazards assumption was violated, either by statistical testing or visual inspection of KM curves. Finally, the “survminer” package (version 0.5.0; Kassambara Lab) was used to generate and visualize the KM plots.^29^

NMA was conducted using a frequentist framework^30^ implemented in the “netmeta” package (version 3.2-0; University of Freiburg).^31^ Secondary outcomes, including time to return to normal activities and VCSS score at 6 months, were summarized as pooled mean difference (MD). All models were estimated using the DerSimonian-Laird random-effects method. *P* scores were used to rank interventions according to their relative performance in the NMA, ranging from 0 to 1. A higher *P* score indicates a greater probability that an intervention performs better than the other competing interventions for the specified outcome, based on the estimated treatment effects and their uncertainty.^32^ Publication bias was evaluated both quantitatively and qualitatively using Egger test and inverted funnel plots, respectively.^33^ Heterogeneity was quantified using Higgins' *I*^2^ statistic.^34^ Inconsistency, defined as disagreement between direct and indirect evidence for the same treatment comparison in an NMA, was assessed using both local and global approaches: the “separating indirect from direct evidence” method for local inconsistency, and Cochran's *Q* statistic for global inconsistency.^35^, ^36^, ^37^, ^38^ Subgroup analyses were performed by stratifying the NMA according to continent and type of analysis, with formal tests of interaction used to assess whether treatment effects differed across these subgroups. Network meta-regression was performed using a consistency model, which assumes agreement between direct and indirect evidence in the network. Independent slopes were used to allow covariates to influence treatment effects differently across comparisons. The “restricted maximum likelihood” method was used under a random-effects framework. For the complication outcome, each study arm contributed a single cumulative complication proportion at the latest reported follow-up, and analyses were conducted at the study level. Intervention-specific proportions were synthesized without between-intervention comparisons using meta-proportion analysis with random effects generalized linear mixed models to account for between-study heterogeneity. Certainty of evidence was appraised using the Grading of Recommendations Assessment, Development and Evaluation (GRADE) approach, considering six domains: within-study bias (based on RoB-2), reporting bias, indirectness, imprecision, heterogeneity, and incoherence.^39^, ^40^, ^41^

## Results

### Overview of the study selection process

**Fig 1:**
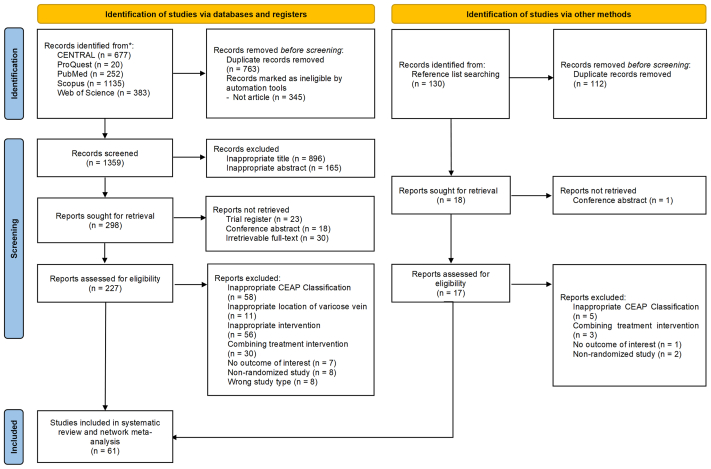
Preferred Reporting Items for Systematic Reviews and Meta-Analyses (PRISMA) flow diagram of the study selection process. *CEAP,* Clinical-Etiology-Anatomy-Pathophysiology classification; *CENTRAL,* Cochrane Central Register of Controlled Trials.

Initial database searches resulted in 2467 records. After removing duplicates and nonarticle records, the remaining 1359 records were screened based on title and abstract, followed by excluding trial registers, conference abstracts, and reports with irretrievable full-text. We thoroughly screened the full-text of 227 reports, and subsequently excluded 178 reports that did not meet the eligibility criteria (Fig 1). In addition, reference list searching resulted in the identification of six eligible studies. Finally, 47 reports^42^, ^43^, ^44^, ^45^, ^46^, ^47^, ^48^, ^49^, ^50^, ^51^, ^52^, ^53^, ^54^, ^55^, ^56^, ^57^, ^58^, ^59^, ^60^, ^61^, ^62^^,^^63^, ^64^, ^65^, ^66^, ^67^, ^68^, ^69^, ^70^, ^71^, ^72^, ^73^, ^74^, ^75^, ^76^, ^77^, ^78^, ^79^, ^80^, ^81^, ^82^, ^83^, ^84^, ^85^, ^86^, ^87^, ^88^ from 33 RCTs were included in this NMA.

### Characteristics of included studies

The inclusion and exclusion criteria for each study are provided in Supplementary Table III (online only). The details of treatment modalities and study characteristics are presented in Supplementary Tables IV and V (online only), respectively. From 33 RCTs, a total of 7913 GSV insufficiency patients in baseline data were accumulated, with a mean age ranging from 34 to 61 years. Female participants accounted for more than 50% of the study population in almost all studies. Most studies were conducted in Europe (n = 19), followed by Asia (n = 12), America (n = 7), and Africa (n = 1). Other two studies were conducted in both America and Europe. Treatment modalities across studies included EVLA-810 nm (n = 5), EVLA-940 nm (n = 2), EVLA-980 nm (n = 7), EVLA-1470 nm (n = 9), RFA (n = 19), MOCA (n = 2), electrocoagulation ablation (n = 1), CAC (n = 5), UGFS (n = 8), and HL&S (n = 18). Adherence rates were excellent across studies, with an average rate of 90.06%. During follow-up, 49 deaths were reported among 7913 patients across the included studies, yielding an overall mortality rate of 0.619%. Supplementary Table VI (online only) provides other baseline patient characteristics. Details of CEAP class were available in 25 studies involving 4807 patients, with C2 class diagnosed in 2596 patients, C3 in 1470 patients, C4 in 683 patients, and C5 in 58 patients. Diameter of treated veins varied from 5.53 to 11.45 mm, whereas length of treated veins varied from 26.15 to 45.10 cm.

### Quality assessment of included studies

Results of quality assessment using RoB-2 tool showed that 16 studies had a low-bias risk, 12 had some concerns, and 5 had a high-bias risk. Viewing results from each domain using a summary bar plot (Supplementary Fig 1, online only), concerns of bias risk most commonly arose from the measurement of the outcome and the randomization process, since a number of studies did not provide information regarding the study protocol and/or did not clearly describe the methods of randomization. Results of quality assessment for each study are provided in Supplementary Fig 2 (online only).

### Time-to-treatment failure

**Fig 2:**
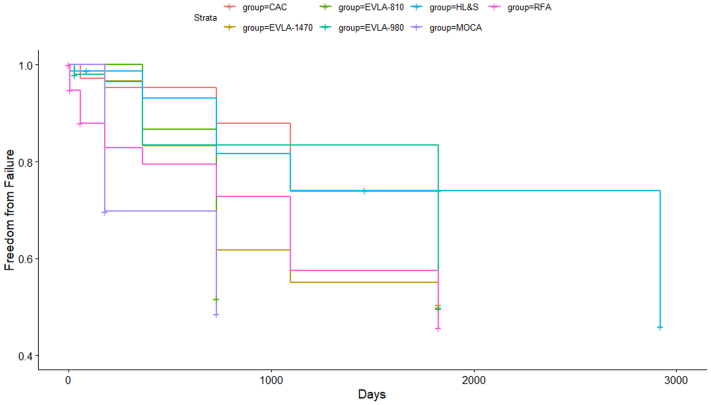
Pooled Kaplan-Meier curves showing the cumulative freedom from failure across time.

**Fig 3:**
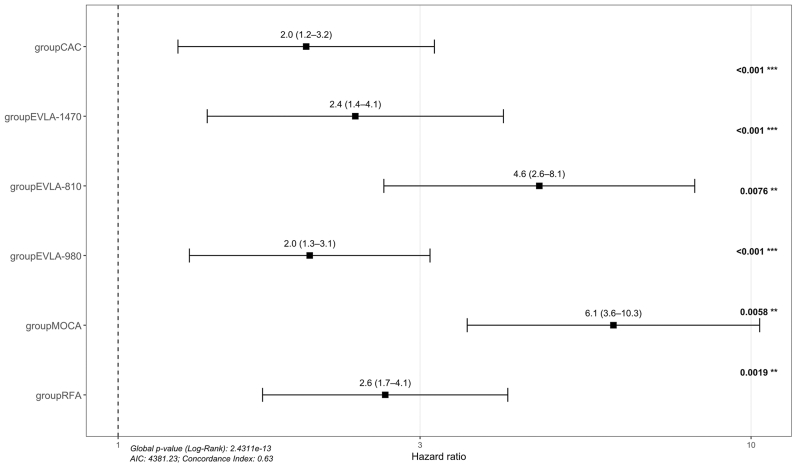
Forest plot of Cox-proportional hazards model for freedom from failure across time. *Error bars* represent Holm-Bonferroni-adjusted 95% confidence intervals. *P* values shown are adjusted using the Holm-Bonferroni method to control the family-wise type I error rate. The global *P* value was obtained from the log-rank test. Model performance is summarized using the Akaike Information Criterion (*AIC*) and the concordance index (*C-index*).

Fig 2 presents the pooled KM curve for cumulative freedom from failure across all included interventions, derived from 33 RCTs. The overall log-rank test demonstrated significant differences in time-to-treatment failure among groups (*χ*^*2*^ = 74.2; *P* < .001). Within the pooled-KM, HL&S, RFA, CAC, and EVLA-1470 nm were the most frequently reported interventions, providing a larger amount of time-to-event data compared with other interventions. The Cox-proportional hazards model showed that CAC (HR, 1.98; 95% CI, 1.24-3.16; *P* < .001), EVLA-1470 nm (HR, 2.37; 95% CI, 1.38-4.06; *P* < .001), and RFA (HR, 2.64; 95% CI, 1.69-4.12; *P* = .002) were all associated with a significantly higher risk of failure compared with HL&S. The forest plot of Cox model is shown in Fig 3. Other interventions included in the pooled KM: EVLA-980 nm, EVLA-810 nm, and MOCA were less frequently reported. Interestingly, these interventions, EVLA-980 nm (HR, 2.01; 95% CI, 1.29-3.11; *P* < .001), EVLA-810 nm (HR, 4.62; 95% CI, 2.63-8.15; *P* = .007), and MOCA (HR, 6.06; 95% CI, 3.56-10.32; *P* = .006) also demonstrated a significantly higher risk of failure than HL&S.

### Time to return to normal activities

**Fig 4:**
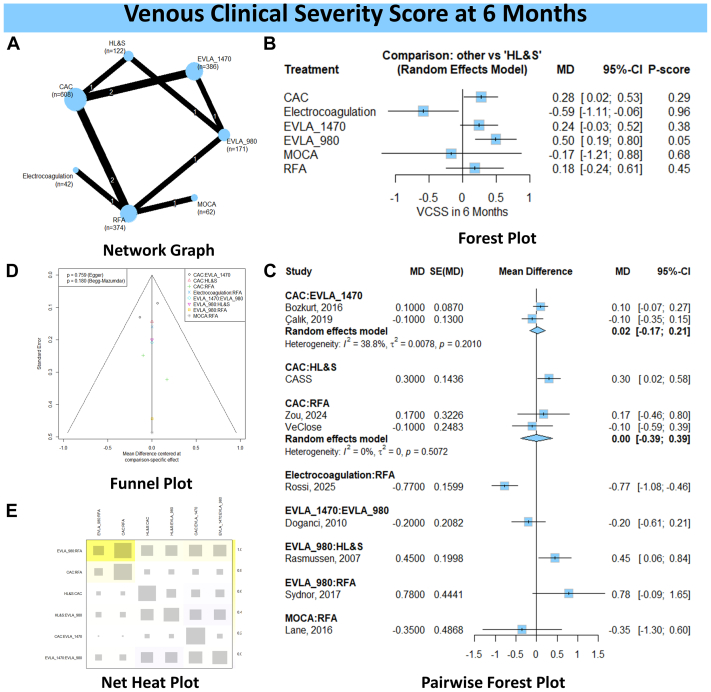
Summary results of frequentist network meta-analysis of time to return to normal activities outcomes. **A,** Network Graph, the width of the line indicates the number of direct pairwise comparisons. The width of the node indicates the number of samples contained in the treatment; **B,** Forest plot; **C,** Pairwise forest plot, treatment effects are expressed as mean differences with corresponding 95% confidence intervals (*CIs*). High ligation and stripping (*HL&S*) used as the reference treatment. Negative mean difference (*MD*) values indicate shorter time to return to normal activities compared with HL&S and vice versa. *P* scores represent the relative ranking of treatments within the network meta-analysis, with higher values indicating better performance; **D,** Funnel plot, each point represents an individual study, plotted according to its effect estimate and corresponding standard error. Symmetry of the plot suggests a low risk of small-study effects; **E,** Net heat plot, a quadratic heatmap with an “ash box” in the middle, indicating the importance of a treatment comparison to estimating other treatment comparisons. The colored background indicates the inconsistency of the treatment in the row that can contribute to the treatment in the column.

Fig 4 summarizes the results of the NMA for time to return to normal activities. The NMA included 1512 patients with GSV insufficiency treated with various interventions (Fig 4, *A*). Forest plot (refer to Fig 4, *B*) demonstrated that most minimally invasive interventions were associated with significantly shorter recovery times compared with HL&S (*P* score = 0.009). Specifically, the following interventions showed a significantly faster return to normal activities: electrocoagulation (MD, −5.29; 95% CI, −9.09 to −1.50; *P* = .006; *P* score = 0.715), EVLA-1470 nm (MD, −2.45; 95% CI, −3.52 to −1.39; *P* < .001; *P* score = 0.474), EVLA-810 nm (MD, −8.67; 95% CI, −16.05 to −1.28; *P* = .02; *P* score = 0.85), MOCA (MD, −2.65; 95% CI, −4.03 to −1.27; *P* < .001; *P* score = 0.515), RFA (MD, −2.05; 95% CI, −2.97 to −1.12; *P* < .001; *P* score = 0.331), and UGFS (MD, −10.08; 95% CI, −11.80 to −8.37; *P* < .001; *P* score = 0.947) (Fig 4, *C*). Assessment of publication bias revealed no-significant Egger test (*P* = .367) and symmetrical funnel plot (Fig 4, *D*). However, moderate-to-high heterogeneity was observed across the NMA (*I*^2^ = 75.4%; 95% CI, 50.5%-87.7%). Further exploration illustrated in Supplementary Table VII (online only), indicated that the main sources of heterogeneity were the RFA:HL&S (*I*^2^ = 89.3%) and MOCA:RFA (*I*^2^ = 40.7%) comparisons. To address this, both local and global inconsistency was evaluated. Netsplit forest plot (Supplementary Fig 3, online only) and net heat plot (Fig 4, *E*) demonstrated no evidence of local inconsistency, whereas global inconsistency (*Q* = 3.59; *P* = .17) likewise indicated no potential of effect modifier within the NMA model. Lastly, the proportion of direct and indirect evidence supporting each intervention comparison is presented in Supplementary Fig 4 (online only).

### Venous clinical severity score at 6 months

**Fig 5:**
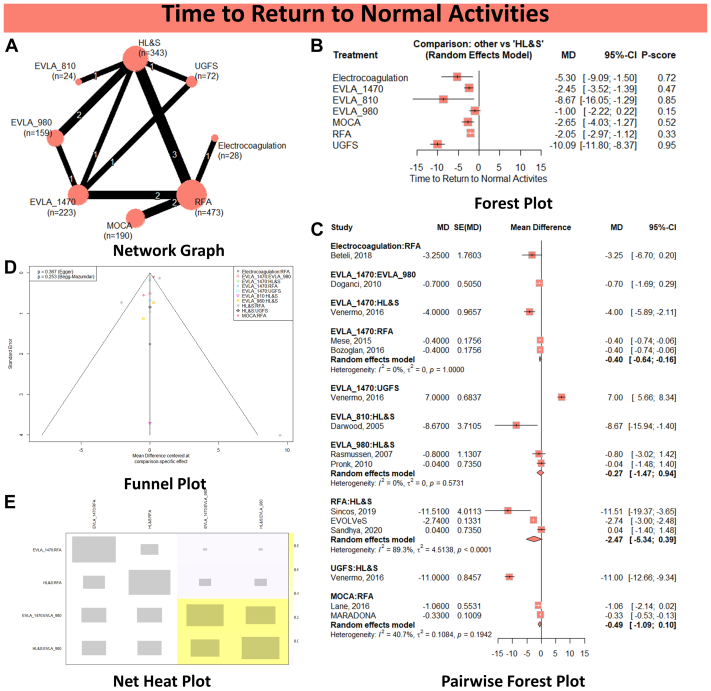
Summary results of network meta-analysis of Venous Clinical Severity Score (VCSS) at 6 months outcomes. **A,** Network graph, the width of the line indicates the number of direct pairwise comparisons. The width of the node indicates the number of samples contained in the treatment; **B,** Forest plot; **C,** Pairwise forest plot, treatment effects are expressed as mean differences with corresponding 95% confidence intervals. High ligation and stripping (*HL&S*) used as the reference treatment. Negative mean difference (*MD*) values indicate lower VCSS at 6 months compared with HL&S and vice versa. *P* scores represent the relative ranking of treatments within the network meta-analysis, with higher values indicating better performance; **D,** Funnel plot, each point represents an individual study, plotted according to its effect estimate and corresponding standard error. Symmetry of the plot suggests a low risk of small-study effects; **E,** Net heat plot, a quadratic heatmap with an “ash box” in the middle, indicating the importance of a treatment comparison to estimating other treatment comparisons. The colored background indicates the inconsistency of the treatment in the row that can contribute to the treatment in the column.

**Fig 6:**
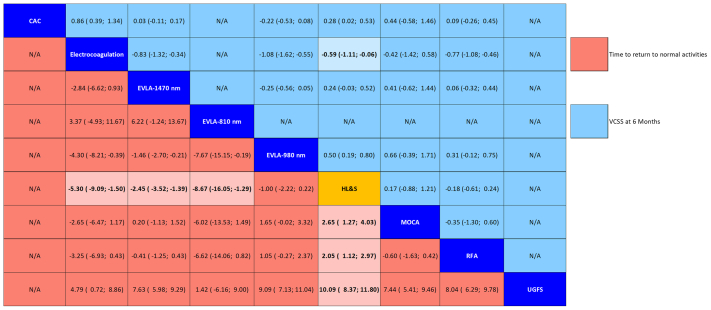
League table of frequentist random-effects model for time to return to normal activities and Venous Clinical Severity Score (*VCSS*) at 6 months outcomes. Comparison should be read from left to right, with outcome estimates shown at the intersection of the column-defining treatment and the row-defining treatment. Results are presented as mean difference [95% confidence interval (*CI*)]. For all outcomes, values below 0 indicate a favorable effect. Interventions are listed in alphabetical order, with high ligation and stripping (*HL&S*) as the reference intervention. The highlighted column indicates treatments demonstrating better outcomes compared with HL&S.

Fig 5, *A* presents the network graph of the NMA for VCSS at 6 months, encompassing 1765 patients with GSV insufficiency. The NMA results (Fig 5, *B*) demonstrated that electrocoagulation (MD, −0.59; 95% CI, −1.11 to −0.06; *P* = .029; *P* score = 0.96) was the only intervention associated with a significantly lower VCSS at 6 months compared with HL&S (*P* score = 0.689). Although MOCA (MD, −0.17; 95% CI, −1.21 to 0.88; *P* = .755; *P* score = 0.678) also showed lower VCSS than HL&S, the difference was not statistically significant (Fig 5, *C*). Interestingly, the treatment ranking placed HL&S (*P* score = 0.689) higher than MOCA (*P* score = 0.678). The funnel plot showing a symmetrical distribution of studies and confirmed by nonsignificant Egger test result (*P* = .759), indicating no potential of publication bias (Fig 5, *D*). Heterogeneity analysis showed negligible to low heterogeneity across the NMA (*I*^2^ = 0%; 95% CI, 0.0%-79.2%), with the primary source identified as the CAC:EVLA-1470 nm comparison (*I*^2^ = 38.8%). Evaluation of local inconsistency using netsplit forest plot (Supplementary Fig 5, online only) and netheat plot (Fig 5, *E*) found no evidence of inconsistency, whereas global inconsistency analysis (*Q* = 1.46; *P* = .48) similarly confirmed the absence of inconsistency within the model. Results of both NMA were further summarized in a league table (Fig 6). The relative contributions of direct and indirect evidence in this NMA are illustrated in Supplementary Fig 6 (online only).

### Complications

Table II summarizes the complications associated with interventions for GSV insufficiency, most of which were minor. Meta-proportions analyses indicated that patients treated with HL&S, had a higher proportion of infection (Prop = .024; 95% CI, 0.015-0.037) and ecchymosis (Prop = .764; 95% CI, 0.424-0.934) compared with other interventions. Notably, bleeding was reported exclusively in patients undergoing HL&S at a proportion of 0.018 (95% CI, 0.005-0.069). In contrast, skin pigmentation was more frequently observed in patients underwent either MOCA (Prop = .114; 95% CI, 0.066-0.191) or UGFS (Prop = .181; 95% CI, 0.042-0.526). Although less commonly reported, burns were associated with both EVLA (Prop = .05; 95% CI, 0.013-0.179), and RFA (Prop = .005; 95% CI, 0.077-0.127) procedures.

**Table II:** Summary of meta-proportions on complications from different interventions for great saphenous vein (GSV) insufficiency

Complications	EVLA			RFA			HL&S		
	k	Prop	95% CI	k	Prop	95% CI	k	Prop	95% CI
Bleeding	–	–	–	–	–	–	2	0.018	0.005-0.069
Burn	2	0.050	0.013-0.179	2	0.005	0.077-0.127	–	–	–
DVT	6	0.008	0.003-0.022	6	0.007	0.002-0.021	8	0.007	0.003-0.016
Dysesthesia	2	0.043	0.030-0.060	1	0.055	0.039-0.078	1	0.068	0.038-0.119
Ecchymosis	6	0.436	0.223-0.676	4	0.193	0.081-0.396	2	0.764	0.424-0.934
Edema	4	0.124	0.038-0.335	2	0.650	0.561-0.730	1	0.094	0.054-0.158
Erythema	2	0.101	0.054-0.183	3	0.029	0.011-0.075	1	0.023	0.003-0.145
Hematoma	6	0.130	0.049-0.299	7	0.061	0.028-0.123	12	0.079	0.041-0.147
Induration	7	0.206	0.197-0.358	5	0.122	0.042-0.304	3	0.109	0.016-0.483
Infection	7	0.007	0.002-0.030	6	0.006	0.002-0.019	10	0.024	0.015-0.037
Pain	3	0.047	0.021-0.101	5	0.091	0.049-0.163	6	0.059	0.017-0.181
Paresthesia	16	0.049	0.029-0.083	11	0.056	0.033-0.094	18	0.066	0.043-0.101
Phlebitis	7	0.095	0.073-0.123	6	0.031	0.015-0.066	4	0.035	0.017-0.073
Skin pigmentation	14	0.043	0.028-0.068	8	0.053	0.031-0.088	7	0.029	0.011-0.079
Thrombophlebitis	2	0.022	0.007-0.069	6	0.031	0.015-0.066	3	0.009	0.001-0.060

Complications	MOCA			UGFS			CAC		
	k	prop	95% CI	k	prop	95% CI	k	prop	95% CI
Bleeding	–	–	–	–	–	–	–	–	–
Burn	–	–	–	–	–	–	–	–	–
DVT	1	0.012	0.002-0.077	2	0.021	0.002-0.165	–	–	–
Dysesthesia	–	–	–	–	–	–	–	–	–
Ecchymosis	–	–	–	–	–	–	1	0.792	0.721-0.849
Edema	–	–	–	–	–	–	1	0.015	0.005-0.046
Erythema	–	–	–	–	–	–	–	–	–
Hematoma	1	0.136	0.082-0.217	2	0.007	0.000-0.835	–	–	–
Induration	2	0.068	0.013-0.289	1	0.908	0.819-0.956	–	–	–
Infection	1	0.000	0.000-1.000	2	0.000	0.000-1.000	1	0.009	0.001-0.063
Pain	1	0.097	0.053-0.171	–	–	–	4	0.017	0.009-0.034
Paresthesia	2	0.006	0.001-0.044	3	0.006	0.001-0.028	5	0.013	0.007-0.025
Phlebitis	1	0.035	0.011-0.102	1	0.040	0.006-0.236	3	0.062	0.038-0.099
Skin pigmentation	3	0.114	0.066-0.191	4	0.181	0.042-0.526	3	0.022	0.011-0.041
Thrombophlebitis	1	0.117	0.067-0.194	1	0.074	0.046-0.116	3	0.030	0.014-0.061

*CAC*, Cyanoacrylate closure; *CI*, confidence interval; *DVT*, deep vein thrombosis; *EVLA*, endovenous laser ablation; *HL&S*, high ligation and stripping; *k*, number of studies included in the analysis; *MOCA*, mechanochemical ablation; *Prop*, pooled meta-proportion of event across studies, calculated by pooling study-level proportions using random-effects model; *RFA*, radiofrequency ablation; *UGFS*, ultrasound-guided foam sclerotherapy.

### Subgroup analysis and network meta-regression analysis

The summary of subgroup analyses for time to return to normal activities and VCSS at 6 months outcomes presented in Table III. Subgroup analyses revealed different continent (*P* = .15), type of analysis (*P* = .68), and RoB (*P* = .99) did not significantly affect the pooled results of the time to return to normal activities NMA. Similar results found for VCSS at 6 months outcomes for continent (*P* = .52), type of analysis (*P* = .51), and RoB (*P* = .59) covariates. Table IV summarize the network meta-regression analyses for each outcome. Table III demonstrated that all covariates, including sample size (*P* = .88), adherence rate (*P* = .92), mean age (*P* = .93), baseline CEAP (*P* = .94), and baseline VCSS (*P* = .77) did not make a significantly difference of the time to return to normal activities pooled result. Moreover, difference on sample size (*P* = .91), GSV diameter (*P* = .80), and gender distribution (*P* = .60) also proved to not make a substantial change of VCSS at 6 months NMA result.

**Table III:** Summary of subgroup analyses from each outcomes

Outcome measure	Variables	Subgroup	N	MD	Lower 95% CI	Upper 95% CI	*P* value
Time to return to normal activities	Continent	Asia	370	−0.36	−1.82	1.10	.15
		Europe	956	−2.67	−4.94	−0.39	
		America	186	6.21	−2.19	14.61	
	Type of analysis	ITT	941	−0.40	−0.64	−0.15	.68
		PP	571	−1.52	−6.92	3.87	
	Risk of bias	Low risk	778	−0.40	−1.13	0.33	.99
		Moderate and high risk	734	−0.40	−3.23	2.45	
VCSS at 6 months	Continent	Asia	1003	0.17	−0.48	0.82	.52
		Europe	302	0.25	−0.32	0.82	
		America	460	−0.10	−0.58	0.39	
	Type of analysis	ITT	1360	−0.10	−0.59	0.39	.51
		PP	405	0.17	−0.46	0.80	
	Risk of bias	Low risk	586	−2.70	−7.09	1.68	.59
		Moderate and high risk	1179	−4.00	−5.99	−2.00	

*CI*, Confidence interval; *ITT*, intention-to-treat; *MD*, mean difference; *n*, number of patients included; *PP*, per protocol; *P* value represents treatment-by-covariate interaction test, assessing whether treatment effects differ significantly across covariates subgroups.

**Table IV:** Summary of network meta-regression analyses from each outcomes

Outcome measure	Covariate	β	Lower 95%CI	Upper 95%CI	*P*-value
Time to return to normal activities	Sample size	0.09	−1.08	1.25	.88
	Adherence rate	0.02	−0.47	0.52	.92
	Mean age	−0.21	−5.11	4.68	.93
	Baseline CEAP	1.9	−52.12	55.92	.94
	Baseline VCSS	−2.08	−15.98	11.81	.77
VCSS at 6 months	Sample size	0.01	−0.16	0.18	.91
	GSV diameter	0.27	−1.91	2.46	.80
	Gender distribution	−0.06	−0.27	0.15	.60

*B*, Beta coefficient; *CEAP*, clinical etiological anatomical and pathophysiological classification; *CI*, confidence interval; *GSV*, great saphenous vein; *VCSS*, Venous Clinical Severity Score.

### Certainty of evidence

Supplementary Figs 7 and 8 (online only) illustrates the contribution of RoB assessment to within-study bias in each outcome. Proportions of studies with low risk, some concerns, and high risk are fairly similar. The indirectness contributions in each outcome also reported in Supplementary Figs 9 and 10 (online only). Finally, all domains are summarized within the GRADE report, which presented in Supplementary Table VIII (online only). The GRADE Report demonstrated various confidence rating for comparisons within the time to return to normal activities outcomes. Meanwhile, VCSS at 6 months outcome showing high certainty of evidence in most of the comparisons within the NMA.

## Discussion

### Main findings

According to current guidelines, procedures for GSV insufficiency can be classified into surgical removal, such as HL&S and ablation. Ablation techniques include thermal modalities, such as EVLA and RFA, as well as nonthermal options, such as MOCA.^8^ Newer approaches, such as sclerotherapy, also has been developed.^89^ This study compared the durability and quality-of-life outcomes among various treatment modalities for GSV insufficiency. KM and log-rank test analyses revealed that HL&S maintained the highest long-term procedural freedom from failure, followed by CAC, RFA, and EVLA-1470 nm. Minimally invasive procedures such as UGFS, EVLA-810 nm, and electrocoagulation were associated with the shortest time to return to normal activities compared with HL&S, offering an advantage in early functional recovery. Analysis of VCSS reduction showed the greatest improvement with electrocoagulation, followed by MOCA, although neither demonstrated a statistically significant difference compared to HL&S.

Our study found that HL&S demonstrated greater long-term durability than EVLA. This observation is supported by a meta-analysis by Pan et al,^90^ which reported higher technical freedom from failure for HL&S at 2-year follow up, attributed to more complete vein removal and fewer procedural failures. These findings may be explained by the fact that the HL&S procedure involves complete removal of the refluxing GSV, whereas other techniques may leave residual segments that can lead to recanalization over time, despite the possibility of neovascularization.^91^ Furthermore, the efficacy of other interventions, particularly endovascular approaches, largely depends on factors such as wavelength, tissue penetration, and energy delivery.^92^, ^93^, ^94^ A previous NMA by Bontinis et al^95^ have studied thermal and nonthermal endovenous ablation treatments. The results of the study are in line with current finding, in which both CAC and RFA showed equivalent results compared with EVLA, highlighting the need to consider multiple modalities when optimizing treatment strategies for GSV insufficiency.

Besides its efficacy, patients undergoing HL&S had the longest time taken to return normal activities compared with other treatment modalities. This is because HL&S more commonly involves the use of general or regional anesthesia.^96^ Following general anesthesia, severe postoperative pain causes delayed mobilization, leading to prolong hospitalization, and prohibiting early hospital discharge.^97^ Ablation procedures use local anesthesia, often with a specific technique, called tumescent anesthesia, allowing ambulatory care.^98^ On the other hand, sclerotherapy and electrocoagulation usually does not require any anesthesia. Patients may immediately continue their activities after the procedure, consequently serving as the procedure with the shortest time for patients to return to activities, in line with our result.^99^

Our result showed that VCSS improvement at 6 months was the most pronounced for patients undergoing electrocoagulation ablation. However, studies involving this procedure was only available from one by Beteli et al,^49^ where the electrocoagulator device used was self-developed by the authors. Following electrocoagulation ablation, the procedure with capability of better improving VCSS was HL&S, which is synthesized from two studies. HL&S allows comprehensive removal of the incompetent vein with lower rates of reoperation and reflux, which is then reflected in the lower VCSS score. In addition, according to the *P* score, MOCA performed similarly compared with HL&S in improving VCSS. A previous NMA which included C6 patients by Bontinis et al^95^ have compared the effectiveness of different ablation modalities on VCSS. Contrary to the current finding, the authors found that MOCA, synthesized from two studies, had the least surface under the cumulative ranking curve value in improving VCSS compared with EVLA, RFA, and CAC. Given the limited studies, the most preferable modality for improving VCSS are yet unclear, suggesting the need for further studies.

The results showed complication profiles of different interventions of varicose veins. HL&S was associated with higher rates of ecchymosis and surgical-site infection incidence than endovenous or catheter-based procedures. Endovenous thermal techniques (such as EVLA and RFA) were characterized mainly by thermal-related complications (eg, burns and superficial phlebitic reaction), and catheter/chemical modalities (such as UGFS, MOCA, and CAC) showed more skin pigmentation and induration. Low reported incidence rates of deep vein thrombosis were seen with all modalities. These broad patterns are linear with previous systematic reviews and clinical guidelines.^8^^,^^100^

Ecchymosis was higher in HL&S group compared with endovenous procedures. The most plausible reason is that HL&S entails larger incisions, tissue dissection, and saphenous trunk and tributary traction; this leads to potential dead space for blood accumulation.^101^ In contrast, endovenous procedures involve percutaneous punctures and tumescent anesthesia that compress perivenous tissue during treatment.^102^ The compressed perivenous tissue reduces subcutaneous bleeding and hematoma formation. Nevertheless, endovenous therapies may induce ecchymosis by microperforations of the vein.^103^

Skin pigmentation noticeable after nonsurgical catheter methods, specifically UGFS (18.1%) and MOCA (11.4%) compared with HL&S (2.9%). The pigmentation results from extravasation of erythrocytes or degradation products of remaining intraluminal blood that invade the dermis. Hemoglobin is degraded to hemosiderin, inducing the brown pigmentation. Foam sclerotherapy generates intraluminal thrombosis of the target vein deliberately; therefore the thrombosis on small perforator pathways enables blood or iron products to enter dermal tissues. That is why postsclerotherapy hyperpigmentation is common after the generation of superficial venous thrombus.^3^ HL&S likely have the lowest pigmentation rates (2.9%) because the incompetent vein is actually removed, eliminating much of the residual venous substrate that become the risk of thrombosis.

Superficial phlebitis is most common with EVLA (9.5%) and CAC (6.2%), more than other methods. This is because EVLA high thermal radiation to the vein wall can cause a local vasculitis-like inflammatory reaction. On the other side, CAC is capable of causing substrate-induced phlebitis or local reaction secondary to cyanoacrylate polymer contact with the vessel wall and adjacent tissues.^104^ Infection rates were minimal across all modalities but relatively higher on HL&S method (2.4%). The larger incisions and gross tissue dissection prone to bacterial contamination and subsequent surgical-site infection.^105^ Whereas endovenous techniques involve fewer invasive procedure, which significantly reduce wound exposure as well as tissue dead space. Prophylactic antibiotics were recommended to reduce serious infection and reduce venous thromboembolism, especially in high-risk individuals (eg, diabetes or high body mass index).^106^

### Strengths and limitations

This NMA has several notable strengths. We identified several previously published network meta-analyses (NMAs), including those by Siribumrungwong et al,^105^ Bontinis et al,^95^ Juhani et al,^107^ and Kolluri et al.^108^ However, our analytical framework differs substantially from these existing NMAs. In our study, we incorporated long-term outcomes analyzed using time-to-event methods. More importantly, our Population, Intervention, Comparison, Outcome, and Study Design framework is distinct: we included only patients with CEAP class C2 to C5 chronic venous insufficiency, compared all available interventional modalities, and exclusively analyzed RCTs.

Nevertheless, certain limitations should be acknowledged. First, the number of studies available for some interventions, such as electrocoagulation and MOCA, was limited, restricts the reliability of the evidence for these modalities. Second, although IPD were not available from each included RCT, we ensured the validity of our approach by applying advanced statistical reconstruction methods that have been previously adopted and validated in the literature.^20^ Third, the timeframe for outcomes assessment varied across the included studies, as outcomes were extracted from time-by-time and final follow-up periods reported by each trial. This heterogeneity in follow-up duration may have influenced the reported outcomes and limits the comparability of clinical and safety outcomes across studies. Forth, only limited number of included studies reporting complication outcomes, which resulted in a small effective sample size for these analyses. Consequently, the pooled estimates, particularly in the per-protocol analysis were associated with wide CIs, reflecting increased statistical uncertainty. Fifth, we acknowledge that outcomes of heat-based endovenous ablation may be influenced by early-generation devices, operator experience, and evolving procedural techniques, which could limit the generalizability of these findings to current practice. Sixth, variations in health care systems, discharge policies, anesthesia protocols, and sociocultural factors across studies may contribute to increased heterogeneity in reported clinical outcomes. Lastly, subgroup and network meta-regression analyses on several potential covariates could not be performed, as stratification would have fragmented the network and resulted in insufficient data to sustain meaningful analyses. Further exploration through subgroup and network meta-regression analyses did not identify any covariates significantly accounting for the variability.

## Conclusions

Our findings indicate that HL&S offer higher long-term procedural freedom from failure and clinical improvement, as reflected by VCSS scores, in patients with CEAP stages C2 to C5 disease. Minimally invasive interventions are associated with faster short-term recovery but demonstrate lower long-term durability. Complication profiles vary across interventions, although each carries a distinct risk profile related to its mechanism of action. HL&S is associated with higher proportions of ecchymosis, bleeding, and infection due to its invasive nature, whereas thermal ablation (such as EVLA and RFA) produces predictable thermal complications such as phlebitis and localized inflammation. Chemical and catheter-based techniques (such as UGFS, MOCA, and CAC) reduce incision-related morbidity but are more often associated with pigmentary changes and induration. Collectively, these findings underscore the need to balance long-term efficacy against patient-centered short-term outcomes when selecting treatment strategies for GSV insufficiency. Future studies should further explore patient preferences and cost-effectiveness among the available treatment modalities to strengthen the interpretation and applicability of the present findings.

## Author Contributions

Conception and design: FA, YS, VV, BW

Analysis and interpretation: FA, YS, VV, BW, JH, AW, JD

Data collection: FA, YS, JH, AW

Writing the article: FA, YS, VV, BW, JH, AW

Critical revision of the article: FA, YS, JD

Final approval of the article: FA, YS, VV, BW, JH, AW, JD

Statistical analysis: FA, VV, BW

Obtained funding: Not applicable

Overall responsibility: FA, YS

## Ethical approval and consent to participate

Not applicable.

## Consent for publication

Not applicable.

## Availability of data and materials

All the analysis and data will be made available and can be requested through the first author (F.M.A.).

## Funding

None.

## Disclosures

The authors declare that they have no competing interests.

## Appendix (online only)

Supplementary docx 1
