# Supplementary material for: Network meta-analysis of randomized controlled trials evaluating long-term outcomes of interventional approaches for great saphenous vein insufficiency
Source: J Vasc Surg Venous Lymphat Disord. 2026 Jun 15;14(5):102553. doi: 10.1016/j.jvsv.2026.102553 (PMC13380533; doi:10.1016/j.jvsv.2026.102553)
Supplement: Supplementary docx 1 [file mmc1.docx]

# **Table S1**. PRISMA NMA Checklist of Items to Include When Reporting A Systematic Review Involving a Network Meta-Analysis

| **Section/Topic** | **Item #** | **Checklist Item** | **Reported on Page #** |
| --- | --- | --- | --- |
| **TITLE** |  |  |  |
| Title | 1 | Identify the report as a systematic review *incorporating a network meta-analysis (or related form of meta-analysis).* | ***1*** |
|  |  |  |  |
| **ABSTRACT** |  |  |  |
| Structured summary | 2 | Provide a structured summary including, as applicable:  **Background:** main objectives  **Methods:** data sources; study eligibility criteria, participants, and interventions; study appraisal; and *synthesis methods, such as network meta-analysis.*  **Results:** number of studies and participants identified; summary estimates with corresponding confidence/credible intervals; *treatment rankings may also be discussed. Authors may choose to summarize pairwise comparisons against a chosen treatment included in their analyses for brevity.*  **Discussion/Conclusions:** limitations; conclusions and implications of findings.  **Other:** primary source of funding; systematic review registration number with registry name. | 2 |
|  |  |  |  |
| **INTRODUCTION** |  |  |  |
| Rationale | 3 | Describe the rationale for the review in the context of what is already known*, including mention of why a network meta-analysis has been conducted.* | ***3*** |
| Objectives | 4 | Provide an explicit statement of questions being addressed, with reference to participants, interventions, comparisons, outcomes, and study design (PICOS). | 3 |
|  |  |  |  |
| **METHODS** |  |  |  |
| Protocol and registration | 5 | Indicate whether a review protocol exists and if and where it can be accessed (e.g., Web address); and, if available, provide registration information, including registration number. | 4 |
| Eligibility criteria | 6 | Specify study characteristics (e.g., PICOS, length of follow-up) and report characteristics (e.g., years considered, language, publication status) used as criteria for eligibility, giving rationale. *Clearly describe eligible treatments included in the treatment network, and note whether any have been clustered or merged into the same node (with justification).* | ***4-5, Table 1*** |
| Information sources | 7 | Describe all information sources (e.g., databases with dates of coverage, contact with study authors to identify additional studies) in the search and date last searched. | 4 |
| Search | 8 | Present full electronic search strategy for at least one database, including any limits used, such that it could be repeated. | 4 |
| Study selection | 9 | State the process for selecting studies (i.e., screening, eligibility, included in systematic review, and, if applicable, included in the meta-analysis). | 4 |
| Data collection process | 10 | Describe method of data extraction from reports (e.g., piloted forms, independently, in duplicate) and any processes for obtaining and confirming data from investigators. | 5-6 |
| Data items | 11 | List and define all variables for which data were sought (e.g., PICOS, funding sources) and any assumptions and simplifications made. | 5-6 |
| **Geometry of the network** | **S1** | Describe methods used to explore the geometry of the treatment network under study and potential biases related to it. This should include how the evidence base has been graphically summarized for presentation, and what characteristics were compiled and used to describe the evidence base to readers. | ***5-6*** |
| Risk of bias within individual studies | 12 | Describe methods used for assessing risk of bias of individual studies (including specification of whether this was done at the study or outcome level), and how this information is to be used in any data synthesis. | 6 |
| Summary measures | 13 | State the principal summary measures (e.g., risk ratio, difference in means). *Also describe the use of additional summary measures assessed, such as treatment rankings and surface under the cumulative ranking curve (SUCRA) values, as well as modified approaches used to present summary findings from meta-analyses.* | 6-8 |
| Planned methods of analysis | 14 | Describe the methods of handling data and combining results of studies for each network meta-analysis. This should include, but not be limited to:   - *Handling of multi-arm trials;* - *Selection of variance structure;* - *Selection of prior distributions in Bayesian analyses; and* - *Assessment of model fit.* | 6-8 |
| **Assessment of Inconsistency** | **S2** | Describe the statistical methods used to evaluate the agreement of direct and indirect evidence in the treatment network(s) studied. Describe efforts taken to address its presence when found. | 6-8 |
| Risk of bias across studies | 15 | Specify any assessment of risk of bias that may affect the cumulative evidence (e.g., publication bias, selective reporting within studies). | **6-8** |
| Additional analyses | 16 | Describe methods of additional analyses if done, indicating which were pre-specified. This may include, but not be limited to, the following:   - Sensitivity or subgroup analyses; - Meta-regression analyses; - *Alternative formulations of the treatment network; and* - *Use of alternative prior distributions for Bayesian analyses (if applicable).* | ***6-8*** |
|  |  |  |  |
| **RESULTS†** |  |  |  |
| Study selection | 17 | Give numbers of studies screened, assessed for eligibility, and included in the review, with reasons for exclusions at each stage, ideally with a flow diagram. | 8-9, **Figure 1** |
| **Presentation of network structure** | **S3** | Provide a network graph of the included studies to enable visualization of the geometry of the treatment network. | ***10-13, Figure 4-5*** |
| **Summary of network geometry** | **S4** | Provide a brief overview of characteristics of the treatment network. This may include commentary on the abundance of trials and randomized patients for the different interventions and pairwise comparisons in the network, gaps of evidence in the treatment network, and potential biases reflected by the network structure. | ***10-13, Figure 4-5*** |
| Study characteristics | 18 | For each study, present characteristics for which data were extracted (e.g., study size, PICOS, follow-up period) and provide the citations. | 9, **Table S3-S6** |
| Risk of bias within studies | 19 | Present data on risk of bias of each study and, if available, any outcome level assessment. | 9, Figure S1-S2 |
| Results of individual studies | 20 | For all outcomes considered (benefits or harms), present, for each study: 1) simple summary data for each intervention group, and 2) effect estimates and confidence intervals. *Modified approaches may be needed to deal with information from larger networks.* | 10-13, **Figure 4-5** |
| Synthesis of results | 21 | Present results of each meta-analysis done, including confidence/credible intervals. *In larger networks, authors may focus on comparisons versus a particular comparator (e.g. placebo or standard care), with full findings presented in an appendix. League tables and forest plots may be considered to summarize pairwise comparisons.* If additional summary measures were explored (such as treatment rankings), these should also be presented. | ***10-13, Figure 4-6*** |
| **Exploration for inconsistency** | **S5** | Describe results from investigations of inconsistency. This may include such information as measures of model fit to compare consistency and inconsistency models, *P* values from statistical tests, or summary of inconsistency estimates from different parts of the treatment network. | ***10-13, Figure 4-5*** |
| Risk of bias across studies | 22 | Present results of any assessment of risk of bias across studies for the evidence base being studied. | 18, Figure S5-6 |
| Results of additional analyses | 23 | Give results of additional analyses, if done (e.g., sensitivity or subgroup analyses, meta-regression analyses*, alternative network geometries studied, alternative choice of prior distributions for Bayesian analyses,* and so forth). | ***14-18, Table 2-4*** |
|  |  |  |  |
| **DISCUSSION** |  |  |  |
| Summary of evidence | 24 | Summarize the main findings, including the strength of evidence for each main outcome; consider their relevance to key groups (e.g., healthcare providers, users, and policy-makers). | 18-21 |
| Limitations | 25 | Discuss limitations at study and outcome level (e.g., risk of bias), and at review level (e.g., incomplete retrieval of identified research, reporting bias). *Comment on the validity of the assumptions, such as transitivity and consistency. Comment on any concerns regarding network geometry (e.g., avoidance of certain comparisons).* | 21 |
| Conclusions | 26 | Provide a general interpretation of the results in the context of other evidence, and implications for future research. | 21-22 |
|  |  |  |  |
| **FUNDING** |  |  |  |
| Funding | 27 | Describe sources of funding for the systematic review and other support (e.g., supply of data); role of funders for the systematic review. This should also include information regarding whether funding has been received from manufacturers of treatments in the network and/or whether some of the authors are content experts with professional conflicts of interest that could affect use of treatments in the network. | ***N.A*** |

# Table S2. Data-Specific Search Strategies

| **Database** | **Search terms** | |
| --- | --- | --- |
| PubMed | #1 | Varicose Veins[MeSH Major Topic] |
|  | #2 | "Varices"[Title/Abstract] OR "varicose vein"[Title/Abstract] OR "Venous reflux"[Title/Abstract] OR "Vein insufficiency"[Title/Abstract] OR "Venous insufficiency"[Title/Abstract] OR "Varicosis"[Title/Abstract] OR "Varix"[Title/Abstract] OR "Venous incompetence"[Title/Abstract] |
|  | #3 | "EVLA"[Title/Abstract] OR "endovenous laser ablation"[Title/Abstract] OR "endovenous ablation"[Title/Abstract] OR "endovenous laser treatment"[Title/Abstract] OR "endovenous laser therapy"[Title/Abstract] OR "EVLT"[Title/Abstract] OR "laser therapy"[Title/Abstract] OR "laser ablation"[Title/Abstract] |
|  | #4 | Radiofrequency Ablation[MeSH Major Topic] |
|  | #5 | "radiofrequency ablation"[Title/Abstract] OR "RFA"[Title/Abstract] |
|  | #6 | "endovenous microwave ablation"[Title/Abstract] OR "EVMA"[Title/Abstract] |
|  | #7 | "Mechanochemical ablation"[Title/Abstract] OR "MOCA"[Title/Abstract] OR "mechanochemical endovenous ablation"[Title/Abstract] |
|  | #8 | "CAC"[Title/Abstract] OR "Cyanoacrylate"[Title/Abstract] |
|  | #9 | "Ultrasound-guided foam sclerotherapy"[Title/Abstract] OR "UGFS"[Title/Abstract] OR "sclerotherapy"[Title/Abstract] |
|  | #10 | "cryostripping"[Title/Abstract] OR "cryo stripping"[Title/Abstract] OR "cryo-stripping"[Title/Abstract] |
|  | #11 | "stripping"[Title/Abstract] OR "high ligation and stripping"[Title/Abstract] OR "HL&S"[Title/Abstract] |
|  | #12 | #1 OR #2 |
|  | #13 | #3 OR #4 OR #5 OR #6 OR #7 OR #8 OR #9 OR #10 OR #12 |
|  | #14 | #12 AND #13 |
|  | #15 | "randomized controlled trial*"[Title/Abstract] OR "RCT"[Title/Abstract] |
|  | #16 | #14 AND #15 |
| Scopus | #1 | TITLE-ABS-KEY ("EVLA" OR "endovenous laser ablation" OR "endovenous ablation" OR "endovenous laser treatment" OR "endovenous laser therapy" OR "EVLT" OR "laser therapy" OR "laser ablation" OR "radiofrequency ablation" OR "RFA" OR "endovenous microwave ablation" OR "EVMA" OR "Mechanochemical ablation" OR "MOCA" OR "mechanochemical endovenous ablation" OR "CAC" OR "Cyanoacrylate" OR "Ultrasound-guided foam sclerotherapy" OR "UGFS" OR "sclerotherapy" OR "cryostripping" OR "cryo stripping" OR "cryo-stripping" OR "stripping" OR "high ligation and stripping" OR "HL&S") |
|  | #2 | TITLE-ABS-KEY ("Varices" OR "varicose vein" OR "Venous reflux" OR "Vein insufficiency" OR "Venous insufficiency" OR "Varicosis" OR "Varix" OR "Venous incompetence") |
|  | #3 | TITLE-ABS-KEY("randomized controlled trial" OR "RCT") |
|  | #4 | TITLE("animal" OR "review" OR "meta-analysis" OR "systematic review" OR "case report" OR "case series") |
|  | #5 | #1 AND #2 AND #3 |
|  | #6 | #5 NOT #4 |
| ProQuest | #1 | noft ("EVLA" OR "endovenous laser ablation" OR "endovenous ablation" OR "endovenous laser treatment" OR "endovenous laser therapy" OR "EVLT" OR "laser therapy" OR "laser ablation" OR "radiofrequency ablation" OR "RFA" OR "endovenous microwave ablation" OR "EVMA" OR "Mechanochemical ablation" OR "MOCA" OR "mechanochemical endovenous ablation" OR "CAC" OR "Cyanoacrylate" OR "Ultrasound-guided foam sclerotherapy" OR "UGFS" OR "sclerotherapy" OR "cryostripping" OR "cryo stripping" OR "cryo-stripping" OR "stripping" OR "high ligation and stripping" OR "HL&S") |
|  | #2 | noft ("Varices" OR "varicose vein" OR "Venous reflux" OR "Vein insufficiency" OR "Venous insufficiency" OR "Varicosis" OR "Varix" OR "Venous incompetence") |
|  | #3 | noft ("randomized controlled trial" OR "RCT") |
|  | #4 | #1 AND #2 AND #3 |
| Web of Science | #1 | ALL=("EVLA" OR "endovenous laser ablation" OR "endovenous ablation" OR "endovenous laser treatment" OR "endovenous laser therapy" OR "EVLT" OR "laser therapy" OR "laser ablation" OR "radiofrequency ablation" OR "RFA" OR "endovenous microwave ablation" OR "EVMA" OR "Mechanochemical ablation" OR "MOCA" OR "mechanochemical endovenous ablation" OR "CAC" OR "Cyanoacrylate" OR "Ultrasound-guided foam sclerotherapy" OR "UGFS" OR "sclerotherapy" OR "cryostripping" OR "cryo stripping" OR "cryo-stripping" OR "stripping" OR "high ligation and stripping" OR "HL&S") |
|  | #2 | ALL=("Varices" OR "varicose vein" OR "Venous reflux" OR "Vein insufficiency" OR "Venous insufficiency" OR "Varicosis" OR "Varix" OR "Venous incompetence") |
|  | #3 | ALL=("randomized controlled trial" OR "RCT") |
|  | #4 | #1 AND #2 AND #3 |
| CENTRAL | #1 | MeSH descriptor: [Varicose Veins] explode all trees |
|  | #2 | ("Varices" OR "varicose vein" OR "Venous reflux" OR "Vein insufficiency" OR "Venous insufficiency" OR "Varicosis" OR "Varix" OR "Venous incompetence"):ti,ab,kw |
|  | #3 | ("EVLA" OR "endovenous laser ablation" OR "endovenous ablation" OR "endovenous laser treatment" OR "endovenous laser therapy" OR "EVLT" OR "laser therapy" OR "laser ablation"):ti,ab,kw |
|  | #4 | MeSH descriptor: [Radiofrequency Ablation] explode all trees |
|  | #5 | ("radiofrequency ablation" OR "RFA"):ti,ab,kw |
|  | #6 | ("endovenous microwave ablation" OR "EVMA"):ti,ab,kw |
|  | #7 | ("Mechanochemical ablation" OR "MOCA" OR "mechanochemical endovenous ablation"):ti,ab,kw |
|  | #8 | ("CAC" OR "Cyanoacrylate"):ti,ab,kw |
|  | #9 | ("Ultrasound-guided foam sclerotherapy" OR "UGFS" OR "sclerotherapy"):ti,ab,kw |
|  | #10 | ("cryostripping" OR "cryo stripping" OR "cryo-stripping"):ti,ab,kw |
|  | #11 | ("stripping" OR "high ligation and stripping" OR "HL&S"):ti,ab,kw |
|  | #12 | #1 OR #2 |
|  | #13 | #3 OR #4 OR #5 OR #6 OR #7 OR #8 OR #9 OR #10 OR #11 |
|  | #14 | #12 AND #13 |
|  | #15 | MeSH descriptor: [Randomized Controlled Trial] explode all trees |
|  | #16 | ("randomized controlled trial" OR "RCT"):ti,ab,kw |
|  | #17 | #15 OR #16 |
|  | #18 | #14 AND #17 |

# **Table S3.** Inclusion and exclusion criteria of included studies.

| **Study** | **Inclusion Criteria** | **Exclusion Criteria** |
| --- | --- | --- |
| VeClose | - Age >=21 years and >=70 years at the time of screening - Reflux in the GSV >0.5 second - One or more of the following symptoms related to the target vein: aching, throbbing, heaviness, fatigue, pruritus, night cramps, restlessness, generalized pain or discomfort, swelling - GSV diameter while standing of 3-12 mm throughout the target vein as measured by duplex ultrasound - CEAP classification of C2 (if symptomatic)-C4b - Ability to walk unassisted - Ability to attend follow-up visits - Ability to understand the requirements of the study and to provide informed consent | - Life expectancy <1 year - Active treatment for malignant disease other than nonmelanoma skin cancer - Symptomatic peripheral arterial disease with ABI <0.89 - Daily use of narcotic or nonsteroidal anti-inflammatory pain medications to control pain associated with GSV reflux - Current, regular use of systemic anticoagulation (eg, warfarin, heparin) - Previous or suspected deep venous thrombosis or pulmonary embolus - Previous superficial thrombophlebitis in the target GSV - Previous treatment of venous disease in target limb, other than spider vein treatment - Known hypercoagulable disorder - Conditions that prevent vein treatment with either RFA or VSCS - Immobilization or inability to ambulate - Pregnant before enrollment - Tortuous GSV, which, in the opinion of the investigator, will limit catheter placement or require more than one primary access site - Aneurysm of the target vein with local vein diameter >12 mm  - Significant, incompetent, ipsilateral small saphenous veins, intersaphenous veins, or anterior accessory GSVs  16. Known sensitivity to cyanoacrylate adhesives  17. Current participation in another clinical study involving an investigational agent or treatment or within the 30 days before enrollment  18. Patients who require bilateral treatment during the next 3 months  19. Patients who require additional ipsilateral treatments on the same leg within 3 months following treatment |
| Mendes, 2016 | Age between 18 and 60 years Clinical, etiologic, anatomic, pathophysiologic (CEAP): clinical grades 2 to 5 (C2-5), primary (Ep), superficial (As) and reflux only (Pr) Duplex scan confirmed primary bilateral GSV insufficiency requiring surgery (insufficiency with reverse venous flow was regarded significant if persisting more than 0.5 seconds in a standing position) Duplex scan confirmed suitability for RFA (see exclusion criteria) Patients able to give informed consent | Varicose veins without GSV insufficiency on duplex scan Previous varicose vein surgery Associated small saphenous vein reflux, duplication of the GSV at the SFJ, deep venous insufficiency or previous DVT on duplex scan GSV diameter <3 mm or >12 mm in the supine position Thrombus in the GSV Patients with a pacemaker or internal defibrillator Concomitant peripheral arterial disease (ankle-brachial pressure index of <0.9) Patients on oral anticoagulants Patients with high blood pressure not controlled by medication Patients with known thrombophilia, cancer or lupus Pregnancy |
| Beteli, 2018 | - adults with primary chronic venous insufficiency - experiencing symptomatic venous reflux and varicosities - exhibiting incompetence of the GSV - reflux duration of ≥0.5 seconds determined via duplex ultrasound examination while standing | - post-thrombotic syndrome - previous treatment of the GSV - symptomatic peripheral arterial disease - a GSV diameter of >12 mm |
| Zou, 2024 | (1) Age 18-75 years, regardless of gender;  (2) Clinical diagnosis of unilateral/bilateral varicose veins of the great saphenous vein of the lower limbs (superficial veins only), and unilateral/bilateral reflux of the great saphenous vein trunk confirmed by venous ultrasound of the lower limbs, and only the great saphenous vein trunk of the lower limb was treated in this surgery (3) Chronic venous disease diagnosis and classification system (Clinical-Etiology-Anatomy-Pathophysiology, CEAP) grade C2-C4b;  (4) Voluntary participation in this study and understanding of all risks and benefits described in the informed consent document. | (1) daily use of narcotics or nonsteroidal anti-inflammatory analgesics to control varicose vein-related pain;  (2) severe liver or kidney dysfunction, alanine aminotransferase (ALT) >3 times the upper limit of normal; creatinine >221 ÿmol/L;  (3) known, uncorrectable bleeding or severe coagulation disease;  (4) target lesion segment diameter of the great saphenous vein <3 mm or >12 mm;  (5) symptomatic peripheral arterial disease (6) target vein varicose vein aneurysm with a local diameter >12 mm;  (7) deep vein thrombosis or pulmonary embolism;  (8) acute systemic infectious disease;  (9) superficial thrombophlebitis;  (10) interventional or surgical treatment of the main trunk of the great saphenous vein of the target limb;  (11) suffering from other diseases that may make the trial difficult or significantly shorten the patient's life expectancy (<1 year), such as tumors, liver disease, heart failure, etc.;  (12) allergy to local anesthetics such as lidocaine or cyanoacrylates;  (13) including but not limited to pelvic tumors, sequelae of deep vein thrombosis, KT syndrome (KTS) or non-primary varicose veins caused by a history of arteriovenous fistula; (14) extremely tortuous great saphenous vein, not suitable for minimally invasive interventional treatment; (15) pregnancy or lactation;  (16) currently participating in other drug or medical device clinical trials;  (17) deemed unsuitable for inclusion by the investigator. |
| Lin, 2007 | (1) primary superficial varicose veins;  (2) deep vein valve function within normal to grade III (Kistner classification) (2);  (3) no history of venous ulcers;  (4) no history of venous disease surgery;  (5) no history of deep vein thrombosis in the lower limbs;  (6) normal or mildly abnormal collateral vein function;  (7) no history of tumors, immune-mediated connective tissue diseases, or other hematological disorders | |
| Liao, 2020 | - Age 18 to 80 years; - CEAP clinical class C2 to C5 - GSV reflux of >0.5 second (diffused throughout the entire GSV) - GSV diameter in the thigh between 5 and 12 mm - Informed consent | - Absence of clinical conditions for surgical treatment - History of DVT - Presentation with acute thrombophlebitis or chronic obstruction of the GSV detected on the duplex ultrasound examination - Previous VV intervention in the same leg - Coagulation disorders - Peripheral artery disease - Life expectancy <1 year |
| Rasmussen, 2007 | Inclusion criteria were varicose veins,  CEAP C2-4EpAs Pr ,  informed consent,  age 18 to 80 years, and  GSV incompetence as defined by reflux time 0.5 seconds by duplex imaging (Hawk 7-10 MHz probe, BK Medical, Herlev, Denmark).  Previous high ligation with a preserved and refluxing GSV to the groin was also permitted. | Exclusion criteria were duplication of the saphenous trunk or an incompetent anterior accessory GSV,  small saphenous vein reflux until 3 months after removal of such vein,  previous deep venous thrombosis,  history of arterial insufficiency or ankle-brachial index 0.9, or both,  axial deep venous insufficiency (femoral or popliteal vein, or both) and tortuous GSV rendering the vein unsuitable for the treatment. |
| ElKaffas, 2011 | Patients with saphenofemoral junction and great saphenous reflux on duplex ultrasound, either in response to Valsalva maneuver or with standing manual compression and release | - deep or superficial venous thrombosis - patients on anticoagulants - patients with concomitant peripheral arterial disease - pacemakers - serious systemic disease - pregnancy - GSV lumen more than 18 mm in the thigh or - extremely tortuous vein |
| Venermo, 2016 | - Patients aged 20–70 years - CEAP class C2–C4 varicoseveins - reflux in the GSV 5–10 mm in diameter | - Peripheral arterial disease - lymphoedema - BMI exceeding 40 kg/m2 - pregnancy - allergy to the sclerosant or lidocaine - severe general illness - malignancy - previous deep vein thrombosis and coagulation disorder |
| Rautio, 2002 | - Patients suitable for day-case surgery - symptomatic, previously untreated, and uncomplicated GSV tributary varicosis - isolated unilateral SFJ and GSV trunk insufficiency | - coagulopathy - multiple, tortuous, and large-diameter (12 mm) GSV trunks |
| Vähäaho, 2019 | - Clinical classification of C2–C4 - Ultrasound-verified reflux in the GSV - Mean GSV diameter in the thigh between 5 and 12 mm - Age 20–75years - Informed consent | - BMI over 40kg/m2 - Peripheral artery disease - Lymphoedema - Pregnancy - Allergy to either the sclerosant or lidocaine - Severe general illness - Malignancy - Previous deep venous thrombosis - Previous varicose vein intervention in the same leg - Coagulation disorders |
| EVOLVeS | Reverse flow in the GSV lasting longer than 0.5 seconds in the standing position, as routinely used for definition of GSV incompetence by all investigators Age between 21 and 80 years CEAP7 clinical classification C2, C3, or C4 Ambulatory status Segmental deep reflux allowable Saphenous vein diameter less than or equal to 1.2 cm in the supine position Availability for follow-up visits at 72 hours, 1 week, 3 weeks, and 4 months | Vein diameter greater than 1.2 cm or less than 0.2 cm Duplication of saphenous trunk or incompetent accessory saphenous branch Small saphenous vein reflux Varices of the thigh Previous deep venous thrombosis Arterial insufficiency (ankle-brachial index [ABI] 0.9) Axial deep venous reflux, from groin through popliteal vein Tortuosity of the GSV segment to be treated, subjectively assessed on the basis of appearance and ultrasound scan as unsuitable for catheterization |
| RELACS Study | • Great saphenous vein (GSV) insufficiency with saphenofemoral incompetence and reflux at least down to the knee level • CVI and/or symptoms caused by GSV incompetence and/or severe clinical findings at risk of varicose vein bleeding, thrombophlebitis or deep vein thrombosis • Age, 18 to 65 years (at randomization) • Performance status (according to the criteria of the American Society of Anesthesiologists, of class I-II) | • Previous surgical interventions in the groin area with the exception of inguinal herniotomy • Anterior or posterior accessory saphenous vein incompetence • Small saphenous vein insufficiency requiring treatment at the same limb • Acute deep venous thrombosis or postthrombotic syndrome • Known thrombophilia associated with a high risk of thromboembolism • Arterial occlusive disease classified as at least Fontaine stage IIA, and/or ankle-brachial index below 0.8 • Active malignant disease (diagnosed during the past 5 years) • Poor compliance or inability to understand the study-related procedures • Women who are pregnant or nursing |
| Flessenkämper, 2013 | Patients aged 18–70 GSV varicose vein stage C2-C4 anatomically suitable for both surgery and laser therapy Vein diameter <16 mm on 5 cm distal to GSV | Previous surgery on GSV |
| Sandhya, 2020 | Clinically symptomatic patients CEAP classes 2–5 Age > 12 and < 70 years | H/o deep vein thrombosis CEAP class 0, 1 and 6 Severe systemic diseases Local site infection Severe systemic infection Peripheral arterial insufficiency (ankle brachial index < 0.8) Pregnancy Previous treatment of varicose vein Allergy to polidocanol Post-thrombotic syndrome Immobility or confinement to bed Diabetic foot Patent foramen ovale on echocardiography Body mass index > 35 Deep vein reflux |
| Rai, 2019 | - primary varicose veins due to incompetent GSV, - symptomatic varices Clinical Etiologic Anatomic Pathophysiologic classes (C2–4EPAsPr), - diameter of the GSV 3 cm below sapheno-femoral junction of 10–20 mm by color Doppler ultrasound, - no duplication of the saphenous vein, - no current or previous history of deep venous thrombosis (DVT), - not taking anticoagulant or antiplatelet medications by the patients, - no indirect GSV, - no varicose vein recurrence | - pregnant patients - died patients after the intervention - do not cooperate for following visits |
| Tofigh, 2020 | - C2,C3,C4 classification - 18-65 years old - SFJ reflux | - Recurrent cases,  - impossible-to-follow-up patients,  - patients with pregnancy,  - active malignancy,  - acute or old DVT,  - an arterial occlusive disease with an ankle-brachial index of below 0.8,  - known thrombophilia or high risk of pulmonary thromboembolism and history of inguinal surgery except for hernia |
| Doganci, 2010 | symptomatic varicose veins | - Patients with a history of previous DVT, - concomitant peripheral arterial disease (ABPI < 0.8),  - difficulty in ambulation,  - pregnant or breast-feeding,  - recurrent varicose veins and those who had reflux in other axial veins (anterior accessory great saphenous vein, small saphenous vein) or perforators |
| CASS | Aged between 18 and 80 years Reflux in the great saphenous vein of > 0.5 s Diameter of the saphenous vein between 2 and 20 mm (standing position) One or more of the symptoms related to the incompetent saphenous vein CEAP classification of C2 through C5 | Previous treatment in the targeted vein segment Tortuous vein in which the delivery catheter cannot be inserted Aneurysm of target-vein segment of > 20 mm Daily use of narcotic or pain medications to control pain associated with reflux Known hypercoagulable disorder Active malignancy Regular or current use of systemic anticoagulation Previous deep vein thrombosis/pulmonary embolism or active acute superficial thrombophlebitis Unable to comply with the schedule and protocol evaluations Unable to ambulate Unable to provide informed consent Currently pregnant or breastfeeding Known sensitivity to cyanoacrylate adhesives Participation in another clinical study that did not reach the primary endpoint within 30 days prior to enrollment |
| RECOVERY | Patients between the ages of 18 and 80 years with incompetent GSVs documented on duplex ultrasound (US; B-mode and color Doppler imaging) were eligible. Reflux was considered significant if reversal of flow was present for more than 0.5 seconds after distal compression in the standing position. | Exclusion criteria consisted of: thrombus in the vein of interest, previous GSV treatment, pregnancy, known malignancy, and use of anticoagulant medication with the exception of lowdose aspirin. |
| MAGNA | - symptomatic primary incompetent GSV at least above the knee with a diameter of >.5 cm  - an incompetent SFJ | - previous treatment of the ipsilateral GSV, - deep venous incompetence or obstruction,  - agenesis of the deep system,  - vascular malformations,  - use of anticoagulation,  - pregnancy,  - heart failure,  - contraindication for one of the treatments (eg, allergy for aethoxysclerol or lidocaine), - immobility,  - arterial insufficiency (defined as an anklebrachial index <0.6),  - age under 18 years, - inability to provide written informed consent |
| Shadid, 2012 | - Patients with primary GSV incompetence - The presence of one or more venous symptoms, in combination with incompetence of the saphenofemoral junction and GSV (measured over a distance of at least 20 cm in the upper leg) and a reflux time of more than 0.5 s - A normal deep venous system on duplex imaging | - Patients with an incompetent deep venous system - Signs of a previous deep venous thrombosis on duplex imaging - An active ulcer or a contraindication to the use of polidocanol |
| Pronk, 2010 | Patients eligible for surgery, who agreed to have local tumescent anaesthesia, andmet with the inclusion criteria, were invited to participate in the study. Inclusion criteria included age > 18 years at randomisation, CEAP (clinical class, etiology, anatomy, pathophysiology) classification C2,12 GSV and sapheno-femoral junction (SFJ) incompetence defined as reflux > 0.5 s seen on DUS imaging with an intrafascial length of at least 15 cm measured from the SFJ downwards, and GSV diameter between 0.3 and 1.5 cm. | Exclusion criteria included previous surgical treatment of the GSV, intrafascial GSV reflux length 15 cm measured from the SFJ downwards, GSV diameter 0.3 or 1.5 cm, pregnancy, immobility, intolerance of lidocaine, active superficial phlebitis, previous or active deep venous thrombosis, deepvenous insufficiency. |
| van der Velden, 2015 | - Adult patients - Symptomatic primary incompetent GSV at least above the knee with a diameter of ≥0.5 cm and with an incompetent SFJ, defined as reflux of ≥0.5 seconds at color duplex ultrasound | - previous treatment of the ipsilateral GSV - deep venous incompetence or obstruction - agenesis of the deep system - vascular malformations - use of anticoagulation - pregnancy - heart failure - contraindication for one of the treatments (eg, allergy for aethoxysclerol or lidocaine) - immobility - arterial insufficiency (defined as an anklebrachial index <0.6) - age under 18 years |
| MARADONA | Patients were included when suffering from GSV incompetence (>3 mm and <12 mm), with a clinical class between C2 and C5 (Clinical, Etiology, Anatomy, and Pathophysiology classification). | Exclusion criteria were an active ulcer, previous surgery or treatment of the ipsilateral GSV, use of oral anticoagulants, pregnancy or lactation, previous deep venous thrombosis, immobilization, contraindication or known allergy to sclerosant, coagulation disorders or increased risk of thromboembolism, severe renal or liver insufficiency, and severe peripheral artery disease. |
| Çalık, 2019 | Patients between 18–75 years with symptomaticvaricose veins CEAP classification between C2–C5 GSV insufficiency 0.5 sec determined by CDUS could come to follow up examinations and were mentally healthy to approve the operation | Saphenous vein duplication or accessory saphenous vein withvenous insufficiency Advanced tortuous GSV Saphenous vein under 3 mm and over 15-mm diameter history of deep venous thrombosis Active thrombophlebitis in deep or superficial veins Arterial insufficiency history or ankle-brachial indexunder 0.9 Significant femoral or popliteal vein insufficiency History of saphenous vein intervention (surgical,thermal or chemical ablation) Hypersensitivity to the CA glue or reaction historywith the past surgeries Cancer Life expectancy under two years |
| Mese, 2015 | Patients presenting to the cardiovascular surgery department between January and December, 2013, and with a saphenous vein diameter of 10 mm or more at the level of the saphenofemoral joint (SFJ) were enrolled | Patients with a saphenous vein diameter less than 10 mm at the SFJ |
| Bozoglan, 2016 | - patients with symptomatic great saphenous vein insufficiency in both lower extremities | - Patients with unilateral vena saphena magna (VSM) insufficiency,  - patients receiving the same technique in both legs, - patients not permitting intervention on both legs in different sessions - saphenous vein diameter less than 5.5 mm at the saphenofemoral junction (SFJ) |
| Bozkurt, 2016 | The inclusion criteria were primary varicosities with C2–C4b patients (clinical, etiological, anatomical and pathophysiological classification (CEAP)) and a sapheno-femoral junction (SFJ) incompetence and GSV reflux lasting longer than 0.5 s on duplex scanning. | The exclusion criteria included a history of deep vein thrombosis, reflux of femoral vein going beyond the knee, hemodynamically significant reflux of the short saphenous or great saphenous anterior accessory vein, congenital vasculopathies, thrombophilia, severe systemic disease, pregnancy, breast feeding, as well as noncompliant patients for followup. GSV diameter > 15 mm was another exclusion criteria. |
| Darwood, 2005 | Consecutive adult patients (aged over 18 years) with symptomatic varicose veins and primary saphenofemoral incompetence were invited to take part in the study. | Patients were not considered for the study if they were taking warfarin or if they were unsuitable for either EVLA (tortuous GSV, large incompetent anterior accessory saphenous vein) or surgery (co-morbidity prohibited general anaesthesia). |
| Lam, 2018 |  |  |
| Sydnor, 2017 | (1) CVI symptoms caused by GSV reflux, defined as reverse flow in the GSV greater than 0.5 s after calf compression in the standing position,  (2) a clinical-etiology-anatomy-pathophysiology (CEAP) clinical class of 2 or greater, and  (3) prior attempt of at least six weeks of compression stockings for conservative management of CVI. | (1) previous vein surgery, EVTA, or phlebectomy in the target extremity (excluding sclerosant injections for spider veins or other superficial cosmetic procedures), (2) active or prior DVT in the target extremity, (3) active or prior hypercoagulability disorder,  (4) patients who are pregnant or breastfeeding,  (5) patients who are non-ambulatory,  (6) age less than 18 years, and  (7) patients who are prisoners. |
| Gale, 2009 | Between June 2006 and May 2008, patients presenting with symptomatic primary venous insufficiency due to GSV incompetence were evaluated for inclusion in this study | The study excluded patients with post-thrombotic venous disease and patients requiring long term therapeutic anticoagulation |

# **Table S4.** Description of treatment modalities in included studies.

| **Study** | **Arm 1** | | | **Arm 2 / 3** | | |
| --- | --- | --- | --- | --- | --- | --- |
|  | **Intervention** | **Device/brand** | **Short Brief (Dimension, duration, power [W])** | **Intervention** | **Device/brand** | **Short Brief (Dimension, duration, power [W])** |
| VeClose | CAC | VenaSeal Sapheon Closure System (VSCS; Sapheon, Inc, Morrisville, NC) | Briefly, with high-resolution ultrasound guidance, a 5F introducer sheath/catheter was advanced to the saphenofemoral junction (SFJ) and positioned 5.0 cm caudal to the SFJ. With proximal GSV compression by the ultrasound probe, two injections of approximately 0.10 mL CA were given 1 cm apart at this location, followed by a 3-minute period of local compression, and then repeated injections and 30-second ultrasound probe and hand compression sequences until the entire length of the target vein segment wastreated | RFA | ClosureFast (Covidien, Mansfield, Mass) | Perivenous TA was delivered to the saphenous compartment surrounding the vein, and the dosage was recorded. Use of reprocessed catheters was not allowed. Double cycles of RF were employed at the first treatment zone near the SFJ in all subjects. |
| Mendes, 2016 | HL&S | N/A | N/A | RFA | Closure™ system, VNUS Medical Technologies, Inc., San Jose, California, USA | 7-cm heating element, maintained at 120°C per segment for a standard length of time |
| Beteli, 2018 | Electrocoagulation ablation | Electrosurgical Generator FX power source (Valley Lab, Boulder, CO) | 60 W for 10 seconds | RFA | Intraluminal Closure FAST catheter | 120°C in 20-second cycles |
| Zou, 2024 | CAC | Shanghai Yisi Miao Medical Devices | Pull the trigger of the injection gun once and hold it for 3 seconds to deliver 0.10 ml (fixed volume) of glue into the vein. Immediately withdraw the locked catheter sheath and injection catheter by 1 cm. Pull the trigger again and hold it for 3 seconds. After the second injection, immediately withdraw 3 cm. Maintain lateral pressure with the ultrasound probe at the glue injection site for at least 3 minutes. Pull the trigger of the injection gun again and hold it for 3 seconds. Immediately withdraw 3 cm after the injection and maintain lateral pressure (using the ultrasound transducer) for at least 30 seconds | RFA | ClosureFast | N/A |
| Lin, 2007 | EVLA 810 nm | DIOMED laser therapy device | 810 nm, 12 - 14 W, withdrawn at a speed of 0.3 - 0.5 cm/s | HL&S | N/A | N/A |
| Liao, 2020 | RFA | Celon RFiTT (Olympus, Tokyo, Japan), an impedance-controlled bipolar radiofrequency catheter that uses a bipolar transducer, generates high-frequency heat waves on the vascular membrane  The TriVex system (Smith & Nephew, Andover, Mass) is made of a central tower that can control a xenon light source, an irrigation pump, and a resector handpiece that is available in two sizes, 4.5 and 5.5 mm | The working segment at the end of the electrode needle is 1.5 cm long and transmits heat generated by high-frequency vibrations to the GSV through direct contact with the vascular endothelium. The working segment is heated to about 60C to 80C and closes the vein rapidly > An average of five to seven probe passages were performed per treated zone at 1 s/cm, and the power was set as 18 W.  The TriVex system (Smith & Nephew, Andover, Mass) is made of a central tower that can control a xenon light source, an irrigation pump, and a resector handpiece that is available in two sizes, 4.5 and 5.5 mm > Several small incisions were made and used for TIPP; lower oscillation frequency (300-400 rpm) was used for suction and morcellation of VVs. | HL&S | No specific device | In the HL&S group, the operation was carried out under general anesthesia through a short incision in the groin. The tributaries of the GSV were ligated, and then the GSV was removed from above-knee level to the groin by use of a pin stripper. In all patients, we first tried to use the inversion stripping technique; if that failed, we used a mushroom tip on the end of the stripper. Additional TIPP procedures are performed the same as in the RFiTT group described before. |
| Rasmussen, 2007 | HL&S | N/A | a groin incision of 4 to 6 cm, with flush division of the GSV and division of all tributaries behind the second level of the division. | EVLA 980 nm | Ceralas D 980, Biolitec | 980-nm diode laser, pulse mode with 1.5-second impulse, 1.5-second pause, and 12 W of energy |
| ElKaffas, 2011 | RFA | Bipolar generator and catheters with sheathable electrodes (Closure system, VNUS Medical Technologies Inc, San Jose´, California) | Baseline impedance kept at 250 to 300 Ω and baseline temperature 32C-37C | HL&S | N/A | Stripping of the vein was done after wrapping elastic bandage to reduce postoperative hematoma and the operation table was tilted 30 foot up |
| Venermo, 2016 | HL&S | N/A | Retrograde invagination stripping of the GSV was done, usually down tobelow the knee. Tumescent solution (450 ml Ringer’s solution with 50ml 1 per cent lidocaine with adrenaline(epinephrine)) was injected into the tunnel of the stripped GSV. After stripping, hook phlebectomies were done through tiny incisions, using tumescent solution to minimize haematoma formation. | EVLA 1470 nm | ELVes®; Biolitec | 1470-nm radial laser, pulsed mode, with a 1,5 s impulse and 12W of energy, applying 70 J/cm GSV |
|  |  |  |  | UGFS | Aetoxysclerol®; Kreussler, Wiesbaden, Germany Fibrovein™; STD Pharmaceutical Products, Hereford, UK | Sclerosant-to-air ratio of 1 : 2 using 1% polidocanol or 1% and 3% sodium tetradecyl sulphate |
| Rautio, 2002 | RFA | VNUS Closure System, VNUS Medical Technologies, Inc, Sunnyvale, Calif | 85° C for 15 seconds | HL&S | Flexible and disposable Venostrip (Aesculap AG & CO, KG, Tuttlingen, Germany) | Flexible and disposable Venostrip with a 9-mm olive |
| Vähäaho, 2019 | MOCA | ClariVein® catheter (Vascular Insights, Madison, Connecticut, USA) | Catheter wire rotation was first activated for 2–3s at the highest speed setting (approximately 3500r.p.m.), creating spasm. With the wire continuing to rotate, infusion of the sclerosant was started simultaneously with catheter pull-back. Liquid sodium tetradecyl sulphate (Sotradecol®; AngioDynamics, Queensbury, New York, USA) at a concentration of 1⋅5 per cent was used as the sclerosant | EVLA 1470 nm | 1470-nm diode radial laser (ELVes®; Biolitec, Bonn, Germany) | Pulse mode comprising a 1⋅5-s impulse and 10 W energy, with a protocol to apply 70 J/cm in the GSV |
|  |  |  |  | RFA | VNUSClosure FAST™ catheter (VNUS Medical Technologies, San Jose, California, USA) | The temperature was maintained at 120∘C for 20s per segment using a thermocouple on the heating element, which provided a feedback loop to the generator during withdrawal |
| EVOLVeS | HL&S | an olive-tipped device or a perforate invagination (PIN) stripper. | For patients randomized to S&L, physicians followed their standard practice, using either an olive-tipped device or a perforate invagination (PIN) stripper. | RFA | Closure catheter and system (VNUS Medical Technologies) | For patients randomized to RFO, the Closure catheter and system (VNUS Medical Technologies) was used according to described methods.4,5 |
| RELACS Study | EVLA 1470 nm | MedArt A/S, Hvidovre, Denmark | 810-nm diode laser; 20 W laser continuous. 20J/cm2 vein surface | HL&S | nonresorbable Ethibond | flush ligation of the SFJ with nonresorbable Ethibond followed by invagination of the GSV below the knee |
| Flessenkämper, 2013 | HL&S | N/A | Stripping using invaginating surgical technique | EVLA 980 nm | Biolitec | 980 nm laser with 30 W of continuous energy |
| Sandhya, 2020 | RFA | radiofrequency ablation probe (Closure Fast™), having a 7cm heating element (VNUS Medical Technologies, USA) | Using a guidewire, a 7F vascular sheath was passed into the GSV and a radiofrequency ablation probe (Closure Fast™), having a 7cm heating element (VNUS Medical Technologies, USA) was advanced under ultrasound guidance up to 2.5cm below the saphenofemoral junction. The vein was then ablated with the temperature at 120 degrees for 20 seconds per segment using a thermocouple on the heating element. | HL&S | N/A | a flexible striper was passed to a point 2.5cm below the saphenofemoral junction. Under tumescent infiltration, a small incision was given 2.5cm below the saphenofemoral junction and the vein was hooked out by palpation of the stripper and visual aid. The GSV was divided after ligature and transfixation sutures inserted using 2/0 Vicryl with no attempt at flush ligation. |
| Rai, 2019 | RFA | 7-cm catheter or heating element (Covidian, Costa Rica) | heat was delivered (120°C) for 20 seconds | UGFS | sclerosing solution (Fibrovein solution, United Kingdom) to air ratio of 1:5 | The injection was done until the foam reached to the point nearly 2 cm below the sapheno-femoral junction |
| Tofigh, 2020 | EVLA 980 nm | 600-micron laser fiber | 980-nm diode laser, with 12 W power | RFA | ClosureFast (ClosureFast, Covidien/Medtronic, CA, USA) | N/A |
| Doganci, 2010 | EVLA 980 nm | 600-mm-diameter laser fibre | 980-nm laser, 15 W | EVLA 1470 nm | 600-mm-diameter laser fibre | 1470-nm laser, 15 W |
| CASS | CAC | VenaSeal system (Medtronic) | After successful access of the target vein and insertion of the guidewire, a delivery catheter was advanced to the saphenofemoral junction and positioned 5.0 cm caudally to the junction. With proximal GSV compression by the ultrasound probe, two injections of ∼0.10 mL of cyanoacrylate glue were administered 1 cm apart at this location, followed by a 3-minute period of local compression, with injections and 30-second ultrasound probe and manual compression sequences repeated until the entire length of the target vein segment had been treated. Target vein occlusion was confirmed using duplex ultrasound. | HL&S | N/A | SS was performed with a horizontal incision in the groin, with division and ligation of the GSV and division of all tributaries. The GSV was removed using an intraluminal stripper. |
| RECOVERY | RFA | intraluminally placed Closure-FAST device with a 7-cm heating element. | RF ablation was performed with an intraluminally placed Closure-FAST device with a 7-cm heating element. After positioning the catheter tip 2 cm from the saphenofemoral junction (SFJ), segmental energy delivery at 120°C was delivered in 20-second cycles. Two cycles were applied to the proximal vein, followed by one cycle to the remaining venous segments. | EVLA 980 nm | 980-nm wavelength in the continuous mode at 12 W | The EVL group was treated with a 980-nm wavelength in the continuous mode at 12 W of power with a linear endovenous energy density of 80 J/cm. |
| MAGNA | EVLA 940 nm | N/A | 940-nm diode laser with 60 J/cm2 | UGFS | 1 cc aethoxysclerol 3%: 3 cc air | Tessari-method |
|  |  |  |  | HL&S | N/A | Flush SFJ ligation was followed by ligation of all tributaries back to the second branch and invaginating stripping of the GSV to knee level |
| Shadid, 2012 | UGFS | Sclerosing foam was prepared with the double-syringe technique, applying a 1:4 ratio of sclerosant:air. One syringe was filled with 1 ml 3percent polidocanol (Aethoxysklerol; Kreussler Pharma, Wiesbaden, Germany) and the other syringe with 4 ml air. Patients were treated in the supine position with an 18-Fr intravenous cannula (B. Braun, Melsungen, Germany), which was inserted just above the knee. The treatment was considered successful when the proximal GSV was completely filled with foam and maximal venospasm was achieved. The majority of patients received an injection of 5 ml or more; 48 patients received less than 5 ml. Elevation of the leg or compression of the saphenofemoral junction was not used. Compression was applied with a foam pad over the treated area and an antiembolism stocking (Brevet TX 10 mmHg;M¨olnlycke Health CareBenelux,Breda,TheNetherlands)for1 week, day and night. A class II elastic stocking (Mediven Plus 23 mmHg;Medi,Bayreuth,Germany) wasprescribed during the day for 6 weeks. | Sclerosing foam was prepared with the double-syringe technique, applying a 1:4 ratio of sclerosant:air. One syringe was filled with 1 ml 3percent polidocanol (Aethoxysklerol; Kreussler Pharma, Wiesbaden, Germany) and the other syringe with 4 ml air. Patients were treated in the supine position with an 18-Fr intravenous cannula (B. Braun, Melsungen, Germany), which was inserted just above the knee. The treatment was considered successful when the proximal GSV was completely filled with foam and maximal venospasm was achieved. The majority of patients received an injection of 5 ml or more; 48 patients received less than 5 ml. Elevation of the leg or compression of the saphenofemoral junction was not used. Compression was applied with a foam pad over the treated area and an antiembolism stocking (Brevet TX 10 mmHg;M¨olnlycke Health CareBenelux,Breda,TheNetherlands)for1 week, day and night. A class II elastic stocking (Mediven Plus 23 mmHg;Medi,Bayreuth,Germany) wasprescribed during the day for 6 weeks. | HL&S | After ligating side branches of the GSV, the saphenofemoral junction was ligated and the GSV divided and stripped to just below the knee using a stripper (MultistripTM; Prodimed, Neuilly en Thelle, France). | After ligating side branches of the GSV, the saphenofemoral junction was ligated and the GSV divided and stripped to just below the knee using a stripper (MultistripTM; Prodimed, Neuilly en Thelle, France). |
| Pronk, 2010 | HL&S | Multistrip Prodimed S.A.S. | High ligation and division of the GSV with inversion stripping | EVLA 980 nm | Biolitec | 980-nm diode laser with 12 W of continuous wave laser energy |
| van der Velden, 2015 | HL&S | N/A | High ligation at the SFJ and closure of the cribriform fascia, followed by invaginating stripping | EVLA 940 nm | N/A | 940-nm diode laser at a rate of 60 J/cm |
|  |  |  |  | UGFS | N/A | 1 ml of 3% polidocanol and 3 ml air (Tessari method) |
| MARADONA | MOCA | ClariVein catheter | The wire was activated for 10 seconds to induce vasospasm. The device was withdrawn manually with a speed of 7 s/cm while the liquid sclerosant was continuously injected using 2 mL of 3% polidocanol for the first 10 to 15 cm and 1.5% polidocanol for the remainder | RFA | VNUS ClosureFAST™ catheter (VNUS Medical Technologies, San Jose, California, United States) | 7-cm-long heating element, Every 20 seconds, a new 7-cm segment of the GSV was treated after pullback. The most proximal segment of the GSV was treated with two cycles as recommended by the manufacturer, A temperature of 120°C is generated in the heating element during a 20 second treatment cycle. |
| Çalık, 2019 | CAC | Turkish Glue Kit (TGK) | 2 cc CA, the catheter was primed by pulling the trigger for 1 s. After priming, the trigger was pulled again and pressed for 5 s. While the trigger was being pulled, the delivery catheter was pulled back 2 cm per second. | EVLA 1470 nm | N/A | 1470-nm radial-tip laser, The EVLA catheter was drawn slowly at the rate of 2.08 ± 0.6 cm/sec. The energy applied to the saphenous vein was 40–120 J/cm (74.86 ± 14.04) with 15 W. |
| Mese, 2015 | EVLA 1470 nm | Biolas - 15D, Del YCHI GMBH | Laser energy was applied by adjusting the laser parameters based on the vein diameter and the depth of the vein beneath the skin (12W, 1.2-1.8 mm/sec retraction speed), higher in those parts close to the SFJ, in pulse mode (0.2 sec interval) | RFA | F-Care Systems NV | applying radiofrequency energy to the saphenous vein in the form of 25 W every 0.5 cm (50 watt/cm) from the distal aspect of the SFJ |
| Bozoglan, 2016 | EVLA 1470 nm | EVLAS Circular-2; FG Group, Ankara, Turkey | 1470 nm with 12W diode laser | RFA | F-Care Systems NV, Antwerp, Belgium | 25W every 0.5 cm from distal aspect of the SFJ |
| Bozkurt, 2016 | EVLA 1470 nm | The Evlas Circular Fiber EVLA kit | 600 mm of radially extending fiber that functioned at a wavelength of 1470 nm with the 6 F introducer kit. | CAC | The VariClose Vein Sealing System | One slow pull of the trigger, while pressing for 5 s, gives 0.3 cc of polymer. Every pull (0.3 cc) should be applied for 10 cm length of the vein |
| Darwood, 2005 | EVLA 810 nm | 810-nm diode laser source (EVLT; Diomed, Andover, Massachusetts, USA) EVLA 2 employed continuous laser energy at 14W power and continuous laser fibre withdrawal (2–3 mm/s). | The GSV was cannulated adjacent to the knee under ultrasonographic guidance and a 5-Fr catheter inserted over a guidewire. The catheter tipwas positioned 1–2 cm distal to the saphenofemoral junction (SFJ) and perivenous tumescent anaesthesia achieved by infiltrating 100–150 ml 0·1 per cent lidocaine along the length of the vein under ultrasonographic control. A 600- μmlaser fibre was inserted into the catheter, whichwas then withdrawn by 2 cm to allow the fibre to protrude beyond the catheter tip.The laser was fired as the fibre and catheter were withdrawn simultaneously. | HL&S | perforation invagination stripper (Oesch, Pasli`eres, France) | underwent saphenofemoral ligation, GSV stripping to knee level and multiple phlebectomies of varicosities as a day-case procedure under general anaesthesia. Prophylactic lowmolecular weight heparin was given before surgery. Following a groin crease incision, flush saphenofemoral ligation was performed with ligation of tributaries and closure of the cribriform fascia. Inversion GSV stripping to the kneewas done with a perforation invagination stripper (Oesch, Pasli`eres, France) and phlebectomieswith a vein hook. |
| Lam, 2018 | UGFS | Aethoxysklerol® (Kreussler Pharma, Wiesbaden, Germany) | 1-ml solution mixed with 4 ml air using a double-syringe technique | HL&S | Multistrip™; Prodimed, Neuilly en Thelle, France | Incision in the groin and the saphenofemoral junction (SFJ) was dissected. After ligation of the tributaries of the GSV, the SFJ was flush ligate and the GSV then removed to just below the knee using a stripper |
| Sydnor, 2017 | EVLA 980 nm | Angiodynamics, Queensbury, NY | 980 nm diode laser system at a fluence range of 50 to 80 J/cm and a power setting of 10 W with a constant continuous pullback velocity | RFA | VNUS ClosureFASTTM technology | Heat energy (120C) was then delivered segmentally in discrete 20-s cycles spaced 6.5 cm apart |
| Gale, 2009 | EVLA 810 nm | AngioDynamics Inc | 810-nm diode laser fiber system, 14 W continuous energy, and the fiber was withdrawn at 1 mm/s for the first 10 cm and then at 2.5 to 4.0 mm/s for the remaining distance of the treated vein. | RFA | ClosurePLUS System | 2 cm/min for the first minute of treatment and thereafter at approximately 3 cm/min at temperatures of 85° to 90° C |
| **CAC**, cyanoacrylate closure; **CDFS**, catheter-directed foam sclerotherapy; **EVLA**, endovenous laser ablation; **GSV**, great saphenous vein; **MOCA**, mechanochemical ablation; **N/A**, not available; **RFA**, radiofrequency ablation; **UGFS**, ultrasound-guided foam sclerotherapy. | | | | | | |

# **Table S5.** Basic characteristics of the included studies.

| **Study** | **Trial identifier** | **Study location (country, continent)** | **Sample size (Baseline)** | | **Participants dropped-out** | **Adherence rate (%)** | **% Female (Baseline)** | **Age (Baseline) (years)** | **Analysis type** | **Follow-up assessment tool** | **Point of follow-up** |
| --- | --- | --- | --- | --- | --- | --- | --- | --- | --- | --- | --- |
|  |  |  | **Arm 1 (Baseline)** | **Arm 2 / Arm 3 (Baseline)** |  |  |  |  |  |  |  |
| VeClose | N/A | US, America | 108 | 114 | 10 | 95.5 | 79.28 | 49.77 ± 11.05 | ITT | VCSS, AVVQ, EQ-5D TTO, complication | Day 3, Month 1, Month 3, Month 12, Month 24, Month 36, Month 60 |
| Mendes, 2016 | NCT02588911. | Brazil, America | 18 | 18 | 16 | 55.56 | 61.11 | 49.47 ± 10.17 | PP | DUS, intensity of hyperpigmentation, extension of hematoma, aesthetic results, pain levels, severity of skin burns, nerve injury, and thrombophlebitis | 1. Day 7 2. Day 30 3. Day 180 4. Day 365 |
| Beteli, 2018 | NCT02139085 | Brazil, America | 28 | 29 | 0 | 100 | 72 | 49.54 ± 12.49 | PP | DUS, VCSS, AVVQ | 1 week 3 mo 6 mo |
| Zou, 2024 | ChiCTR2200064862 | China, Asia | 89 | 88 | 10 | 94,35 | 61,58 | 57.63 ± 13.62 | PP | DUS, VCSS, AVVQ | Day 30 Day 90 Day 180 |
| Lin, 2007 | N/A | China, Asia | 40 | 40 | 0 | 100 | 52.5 | 46.86 ± 8.28 | ITT | DUS, APG, VAS | 1. Day 30 2. Day 180 3. Day 365 |
| Liao, 2020 | N/A | China, Asia | 100 | 100 | 14 | 93 | 43.5 | 60.8 ± 12.72 | PP | VCSS, CIVIQ-14, complication | 12 month |
| Rasmussen, 2007 | ISRCTN16747172 | Denmark, Europe | 59 | 62 | 18 | 85.12 | 69.42 | 53.14 ± 11.72 | ITT | VCSS, AVVSS, SF-36, DUS | 1. Day 12 2. Day 30 3. Day 90 4. Day 180  5. 2 year  6. 5 year |
| ElKaffas, 2011 | N/A | Egypt, Africa | 90 | 90 | 18 | 90 | 51.67 | 34 ± 3.31 | PP | DUS | Immediate 7 days 1 month 6 months 1 year 18 months 2 year |
| Venermo, 2016 | N/A | Finland, Europe | 74 | Arm 2: 78 Arm 3: 81 | 27 | 88.41 | 78.5 | 47.6 ± 12.4 | PP | DUS | 52 wk  5 year |
| Rautio, 2002 | N/A | Finland, Europe | 15 | 13 | 0 | 100 | 92.86 | 35.32 ± 7.09 | ITT | DUS | 50 days (mean)  3 year |
| Vähäaho, 2019 | N/A | Finland, Europe | 59 | Arm 2: 34 Arm 3: 32 | 8 | 93.6 | N/A | 50.37 ± 12.4 | PP | AVVQ, VAS, complication | Perioperative, 1 month, 1 year |
| EVOLVeS | N/A | Multicenter, America and Europe | 36 | 44 | 0 | 100.00 | 72.5 | 48.1 ± 4.15 | PP | DUS | 72 hours 1 week  3 week  4 months |
| RELACS Study | ISRCTN18322872 | Germany, Europe | 185 | 160 | 30 | 91.32 | 68.69 | 47.94 ± 10.7 | ITT | DUS | 1. 3 months 2. 12 months 3. 24 months |
| Flessenkämper, 2013 | N/A | Germany, Europe | 159 | 142 | 46 | 84.72 | N/A | N/A | PP | DUS | 2 months 6 months  2 years |
| Sandhya, 2020 | N/A | India, Asia | 33 | 32 | 3 | 95.38 | N/A | 38.95 | PP | DUS | 7 days 1months: 3 months: 1 year |
| Rai, 2019 | N/A | Iran, Asia | 30 | 30 | 5 | 91.67 | 50.91 | 41.89 ± 13.52 | PP | DUS | 1. Day 7 2. 1 month 3. 3 months 4. 6 months |
| Tofigh, 2020 | N/A | Iran, Asia | 545 | 545 | 0 | 100 | 75 | 37.08 ± 9.42 | ITT | DUS | 1. 3 months 2. 6 months 3 . 12 months |
| Doganci, 2010 | N/A | Istanbul, Europe | 30 | 30 | 0 | 100 | 58.33 | 35.5 ± 8.38 | ITT | Clinical examination and DUS | 1. Month 1 2. Months 2 3. Months 3 4. Months 6 |
| CASS | KCT0003203 | Korea, Asia | 63 | 63 | 0 | 100 | 56.35 | 59.8 ± 9.11 | ITT | VCSS, AVVQ, DUS | 1. Day 3 2. Day 30 3. Day 90 4. Day 180 5. Day 360 |
| RECOVERY | N/A | Multicenter, Europe and America | 46 | 41 | 0 | 100 | 78.9 | 51.97 ± 13.95 | PP | DUS | 1 month |
| MAGNA | MEC2005-325 | Netherland, Europe | 80 | Arm 2: 80 Arm 3: 80 | 17 | 92.91 | 67.26 | 52.33 ± 14.86 | PP | N/A | N/A |
| Shadid, 2012 | N/A | Netherland, Europe | 230 | 200 | 40 | 90.7 | 73.02 | 51.18 ± 13.34 | PP | VCSS, EQ-5D, VAS (mean change) | 3, 12, 24 months |
| Pronk, 2010 | N/A | Netherland, Europe | 68 | 62 | 2 | 98.46 | 76.2 | 49.52 ± 10.74 | ITT | DUS | 1 year  5 year  10 year |
| van der Velden, 2015 | NCT00529672  5 year report for study no. 261 | Netherland, Europe | 65 | Arm 2: 70 Arm 3: 64 | 0 | 100 | 69.35 | 52.95 ± 14.75 | PP | DUS, CIVIQ, EQ-5D | 5 year |
| MARADONA | N/A | Netherland, Europe | 105 | 104 | 17 | 91.87 | 60.86 | 50.91 ± 45.16 | ITT | Postprocedural pain, DUS, VCSS, VVQ, SF-36 | Day 14 Day 30 Day 365 Day 730 |
| Çalık, 2019 | N/A | Turkey, Asia | 200 | 200 | 0 | 100 | 55.75 | 38.5 ± 11.84 | ITT | CDUS, physical examination, VCSS, CIVIQ | 1. Day 1 2. Day 7 3. Day 30 4. Day 90 5. Day 180 6. Day 365 |
| Mese, 2015 | N/A | Turkey, Asia | 60 | 60 | 0 | 100 | N/A | N/A | ITT | CDUSG | 1. Day 7 2. Day 30 3. Day 90 4. Day 180 |
| Bozoglan, 2016 | N/A | Turkey, Asia | 60 | 60 | 0 | 100 | 46.67 | 42.2 ± 10.2 | ITT | CDUSG, and clinically | 6 months |
| Bozkurt, 2016 | N/A | Turkey, Asia | 156 | 154 | 27 | 91.29 | 50.97 | 41.34 ± 12.22 | ITT | DUS, VCSS, AVVQ, adverse events | 1. Day 3 2. Day 30 3. Day 180 4. Day 360 |
| Darwood, 2005 | ISRCTN99270116 | United Kingdom, Europe | 33 | 34 | 11 | 90.35 | 56.8 | 45.96 ± 16.76 | PP | DUS | 3 mo 1 year |
| Lam, 2018 | NCT02304146 | US, America | 223 | 227 | 27 | 49.55 | 72.24 | 60.79 ± 11.65 | PP | DUS | 12 months 24 months 36 months 48 months 60 months 72 months 84 months 96 months |
| Sydnor, 2017 | N/A | US, America | 100 | 100 | 47 | 76.5 | 78.5 | 48.40 ± 13.00 | PP | DUS, interview, physical examination, photographs | 1. Day 7 2. Day 42 3. Day 180 4. Day 365 |
| Gale, 2009 | N/A | US, America | 48 | 46 | 12 | 89.83 | 72.03 | 47.53 | PP | DUS, VAS, QoL | 1. Day 7 2. Day 30 3. Day 365 |
| **AVVQ**, Aberdeen Varicose Vein Questionnaire; **AVVSS**, Aberdeen Varicose Vein Symptom Severity Score; **CIVIQ**, Chronic Venous Insufficiency Quality of Life Questionnaire; **DUS**, duplex ultrasound; **EQ-5D**, EuroQol-5 Dimension; **ITT**, intention-to-treat; **N/A**, not available; **PP**, per-protocol; **QoL**, quality of life; **SF-36**, Short Form 36-Item Health Survey questionnaire; **VAS**, Visual Analog Scale; **VCSS**, Venous Clinical Severity Score. | | | | | | | | | | | |

# **Table S6.** Baseline participant characteristics of the included studies.

| **Study** | **CEAP clinical class** | | | | **Total CEAP clinical class** | **AVVSS** | **VCSS** | **AVVQ** | **EQ-5D** | **Diameter of treated GSV (mm)** | **Length of treated GSV (cm)** |
| --- | --- | --- | --- | --- | --- | --- | --- | --- | --- | --- | --- |
|  | **C2** | **C3** | **C4** | **C5** |  |  |  |  |  |  |  |
| VeClose | 125 | 68 | 29 | 0 | N/A | N/A | 5.55 ± 2.59 | 19.15 ± 9.45 | 84.22 ± 14.37 | 6.55 ± 2.3 | 33.48 ± 13.31 |
| Mendes, 2016 | N/A | N/A | N/A | N/A | N/A | N/A | N/A | N/A | N/A | N/A | N/A |
| Beteli, 2018 | 25 | 32 | 24 | 4 | 3.08 ± 0.88 | N/A | 6.6 ± 1.06 | 22.62 ± 4.72 | N/A | 7.75 ± 2.24 | 39.48 ± 6.06 |
| Zou, 2024 | 72 | 50 | 55 | 0 | N/A | N/A | N/A | N/A | N/A | 7.87 ± 2.27 | N/A |
| Lin, 2007 | 19 | 40 | 21 | 0 | N/A | N/A | N/A | N/A | N/A | N/A | N/A |
| Liao, 2020 | 25 | 91 | 91 | 11 | N/A | N/A | RFA: 6.10 ± 2.35 HL&S: 6.22 ± 2.44 | N/A | N/A | RFA: 9.13 ± 2.49 HL&S: 9.29 ± 2.63 | N/A |
| Rasmussen, 2007 | 101 | 8 | 12 | 0 | N/A | 17.89 ± 7.31 | 3.09 ± 1.85 | N/A | N/A | 7.75 ± 2.42 | N/A |
| ElKaffas, 2011 | 96 | 54 | 21 | 9 | 2.68 ± 0.87 | N/A | N/A | N/A | N/A | 7.8 | N/A |
| Venermo, 2016 | 86 | 99 | 29 | N/A | N/A | 31.5 ± 6.9 | N/A | N/A | N/A | N/A | N/A |
| Rautio, 2002 | N/A | N/A | N/A | N/A | N/A | N/A | 5.52 ± 1.44 | N/A | N/A | 6.26 ± 1.51 | N/A |
| Vähäaho, 2019 | 70 | 27 | 27 | 0 | N/A | N/A | N/A | MOCA: 15.8 EVLA: 16.1 RFA: 17.2 | N/A | MOCA: 6.7 ± 1.6 EVLA: 6.5 ± 1.6 RFA: 6.4 ± 1.8 | N/A |
| EVOLVeS | 64 | 8 | 8 | 0 | N/A | N/A | 4.62 ± 0.41 | N/A | N/A | N/A | 38.35 ± 2.49 |
| RELACS Study | 100 | 171 | 71 | 3 | N/A | N/A | N/A | N/A | N/A | 8.7 ± 2.5 | 45.1 ± 13.3 |
| Flessenkämper, 2013 | N/A | N/A | N/A | N/A | N/A | N/A | N/A | N/A | N/A | N/A | N/A |
| Sandhya, 2020 | 14 | 19 | 22 | 4 | N/A | N/A | 6.52 | N/A | N/A | N/A | N/A |
| Rai, 2019 | N/A | N/A | N/A | N/A | N/A | N/A | N/A | 34.08 ± 6.31 | N/A | N/A | N/A |
| Tofigh, 2020 | N/A | N/A | N/A | N/A | N/A | N/A | N/A | N/A | N/A | N/A | N/A |
| Doganci, 2010 | 32 | 59 | 15 | N/A | N/A | N/A | N/A | N/A | N/A | 8.05 ± 2.48 | 39.9 ± 6.64 |
| CASS | 99 | 12 | 10 | 1 | 2.29 ± 0.42 | N/A | 4.7 ± 2.45 | 12.2 ± 8.42 | N/A | 6.25 ± 2.28 | N/A |
| RECOVERY | 79 | N/A | N/A | N/A | N/A | N/A | 4.8 ± 2.96 | N/A | N/A | 5.53 ± 2.55 | 38.65 ± 13.69 |
| MAGNA | 98 | 80 | 30 | 2 | N/A | N/A | N/A | N/A | N/A | N/A | N/A |
| Shadid, 2012 | 359 | 44 | 37 | 11 | N/A | N/A | N/A | N/A | N/A | UGFS Upper thigh: 6.1 ± 2.0 Mid thigh: 5.5 ± 1.8 Lower thigh: 5.2 ± 1.9  HL&S Upper thigh: 6.8 ± 2.4 Mid thigh: 5.9 ± 2.0 Lower thigh: 5.7 ± 2.0 | N/A |
| Pronk, 2010 | 55 | 63 | 9 | 1 | N/A | N/A | N/A | N/A | N/A | 6.4 ± 1.5 | N/A |
| van der Velden, 2015 | 103 | 80 | 30 | 2 | 2.65 ± 0.71 | N/A | N/A | N/A | 0.86 ± 0.18 | 6.24 ± 1.43 | N/A |
| MARADONA | 11 | 132 | 60 | 5 | N/A | N/A | N/A | N/A | N/A | 6.70 ± 9.03 | 38.34 ± 50.52 |
| Çalık, 2019 | 288 | 96 | 16 | 0 | N/A | N/A | 5.75 ± 2.00 | N/A | N/A | 5.8 ± 1.84 | 30.8 ± 5.72 |
| Mese, 2015 | N/A | N/A | N/A | N/A | 3.4 ± 0.80 | N/A | 11.2 ± 2.08 | N/A | N/A | 11.45 ± 1.18 | 26.15 ± 4.53 |
| Bozoglan, 2016 | N/A | N/A | N/A | N/A | 3.2 ± 0.4 | N/A | 9.8 ± 2.5 | N/A | N/A | 8.3 ± 1.89 | 31.95 ± 5.69 |
| Bozkurt, 2016 | 223 | 71 | 16 | 0 | N/A | N/A | 5.7 ± 1.83 | 18.45 ± 4.81 | N/A | 7.15 ± 1.70 | 29.75 ± 6.88 |
| Darwood, 2005 | 84 | 19 | 3 | 4 | N/A | 14.01 ± 7.37 | 4 ± 1.48 | N/A | N/A | N/A | N/A |
| Lam, 2018 | N/A | N/A | N/A | N/A | N/A | N/A | 3.33 ± 2.04 | N/A | N/A | N/A | N/A |
| Sydnor, 2017 | N/A | N/A | N/A | N/A | 2.86 ± 1.62 | N/A | 5.81 ± 4.33 | N/A | N/A | N/A | N/A |
| Gale, 2009 | N/A | N/A | N/A | N/A | N/A | N/A | N/A | N/A | N/A | N/A | N/A |
| **AVVQ**, Aberdeen Varicose Vein Questionnaire; **AVVSS**, Aberdeen Varicose Vein Symptom Severity Score; **CEAP**, Clinical-Etiology-Anatomy-Pathophysiology; **EQ-5D**, EuroQol-5 Dimension; **EVLA**, endovenous laser ablation; **GSV**, great saphenous vein; **MOCA**, mechanochemical ablation; **N/A**, not available; **RFA**, radiofrequency ablation; **UGFS**, ultrasound-guided foam sclerotherapy; **VCSS**, Venous Clinical Severity Score. | | | | | | | | | | | |

**
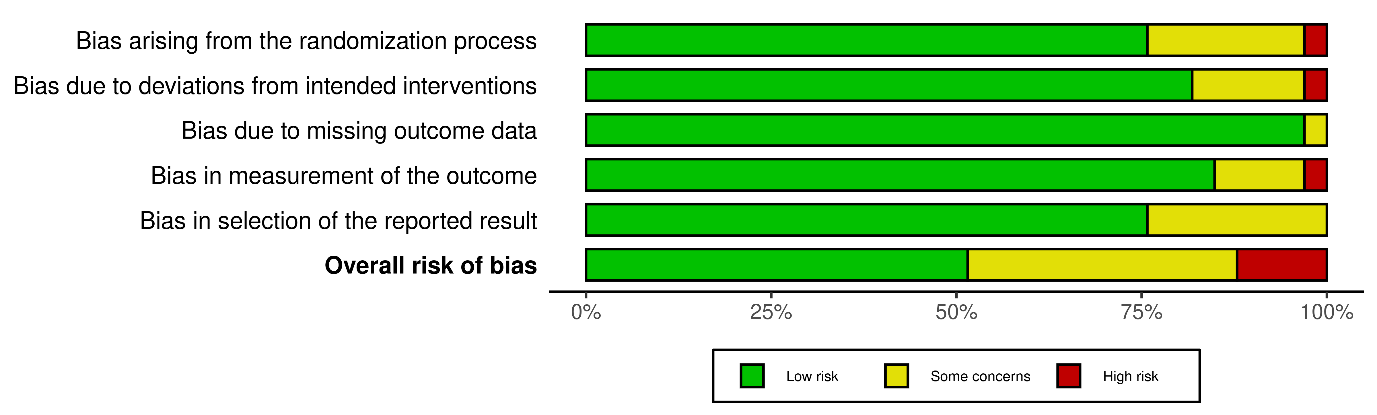
**

# **Figure S1.** Domain-Specific Results of Quality Assessment of Included Studies Using the Risk of Bias 2 Tool


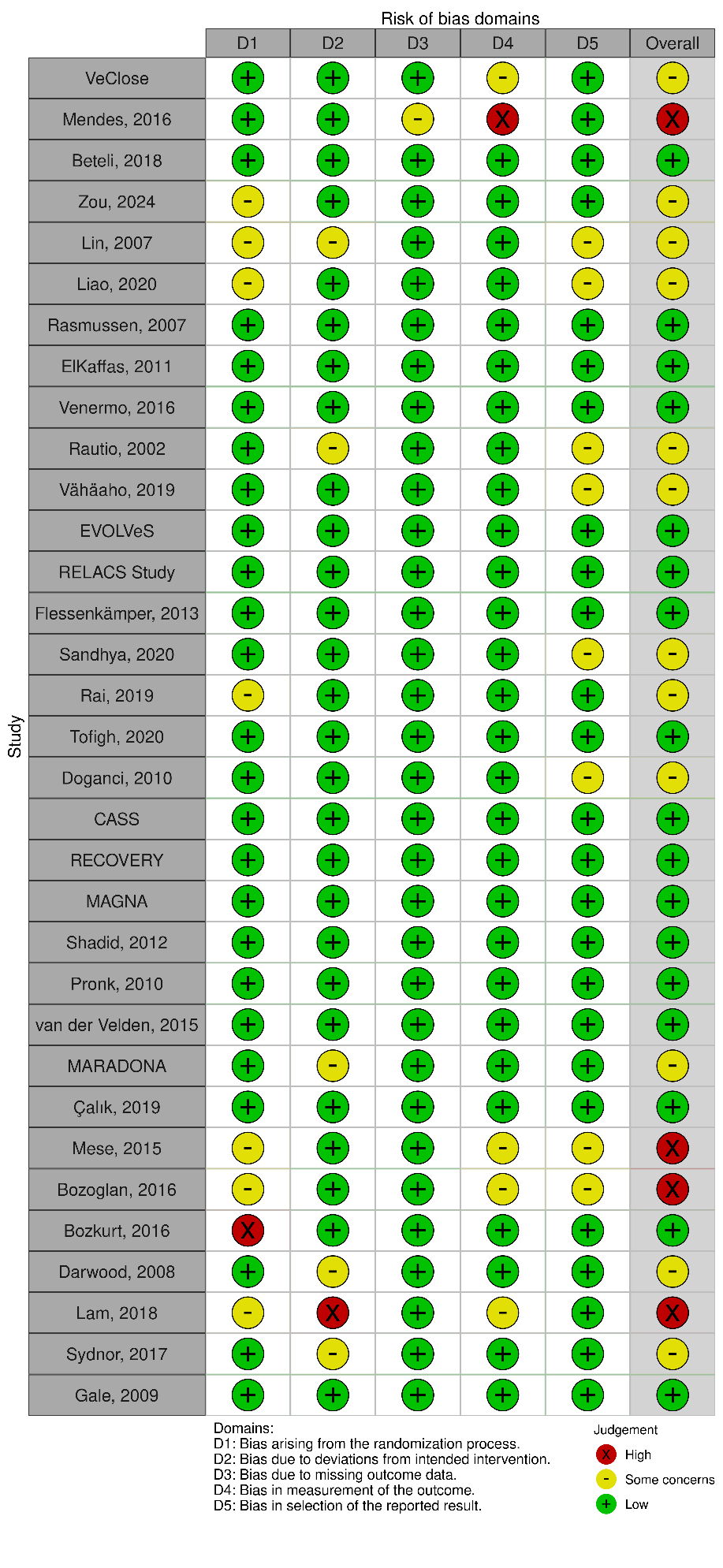


# **Figure S2.** Detailed Quality Assessment Summary of Included Studies Using the Risk of Bias 2 Tool

# **Table S7.** Heterogeneity, Local, and Global Inconsistency Assessment for the Network Meta-Analysis

| **A. Time to Return to Normal Activities** | | | | | | | | |
| --- | --- | --- | --- | --- | --- | --- | --- | --- |
| **Arm 1** | **Arm 2** | **k** | **I^2^** | **Direct estimate** | **Indirect estimate** | **Network**  **meta-analysis** | **Local Inconsistency** | **Global Inconsistency** |
| Electrocoagulation | EVLA_1470 | 0 | N.A | N.A | -2.84 [ -6.62; 0.93] | -2.84 [ -6.62; 0.93] | N.A | *Q* = 3.59 *p* = 0.17 |
| Electrocoagulation | EVLA_810 | 0 | N.A | N.A | 3.37 [ -4.93; 11.67] | 3.37 [ -4.93; 11.67] | N.A |  |
| Electrocoagulation | EVLA_980 | 0 | N.A | N.A | -4.30 [ -8.21; -0.39] | -4.30 [ -8.21; -0.39] | N.A |  |
| Electrocoagulation | HL&S | 0 | N.A | N.A | -5.30 [ -9.09; -1.50] | -5.30 [ -9.09; -1.50] | N.A |  |
| Electrocoagulation | MOCA | 0 | N.A | N.A | -2.65 [ -6.47; 1.17] | -2.65 [ -6.47; 1.17] | N.A |  |
| Electrocoagulation | RFA | 1 | N.A | -3.25 [ -6.93; 0.43] | N.A | -3.25 [ -6.93; 0.43] | N.A |  |
| Electrocoagulation | UGFS | 0 | N.A | N.A | 4.79 [ 0.72; 8.86] | 4.79 [ 0.72; 8.86] | N.A |  |
| EVLA_1470 | EVLA_810 | 0 | N.A | N.A | 6.22 [ -1.24; 13.67] | 6.22 [ -1.24; 13.67] | N.A |  |
| EVLA_1470 | EVLA_980 | 1 | N.A | -0.70 [ -2.32; 0.92] | -2.56 [ -4.52; -0.61] | -1.46 [ -2.70; -0.21] | 0.1504 |  |
| EVLA_1470 | HL&S | 1 | N.A | -4.00 [ -6.28; -1.72] | -2.03 [ -3.23; -0.83] | -2.45 [ -3.52; -1.39] | 0.1341 |  |
| EVLA_1470 | MOCA | 0 | N.A | N.A | 0.20 [ -1.13; 1.52] | 0.20 [ -1.13; 1.52] | N.A |  |
| EVLA_1470 | RFA | 2 | 0.00% | -0.40 [ -1.34; 0.54] | -0.43 [ -2.35; 1.49] | -0.41 [ -1.25; 0.43] | 0.9762 |  |
| EVLA_1470 | UGFS | 1 | N.A | 7.00 [ 5.15; 8.85] | 10.17 [ 6.46; 13.88] | 7.63 [ 5.98; 9.29] | 0.1341 |  |
| EVLA_810 | EVLA_980 | 0 | N.A | N.A | -7.67 [-15.15; -0.19] | -7.67 [-15.15; -0.19] | N.A |  |
| EVLA_810 | HL&S | 1 | N.A | -8.67 [-16.05; -1.29] | N.A | -8.67 [-16.05; -1.29] | N.A |  |
| EVLA_810 | MOCA | 0 | N.A | N.A | -6.02 [-13.53; 1.49] | -6.02 [-13.53; 1.49] | N.A |  |
| EVLA_810 | RFA | 0 | N.A | N.A | -6.62 [-14.06; 0.82] | -6.62 [-14.06; 0.82] | N.A |  |
| EVLA_810 | UGFS | 0 | N.A | N.A | 1.42 [ -6.16; 9.00] | 1.42 [ -6.16; 9.00] | N.A |  |
| EVLA_980 | HL&S | 2 | 0.00% | -0.31 [ -1.85; 1.22] | -2.18 [ -4.19; -0.16] | -1.00 [ -2.22; 0.22] | 0.1504 |  |
| EVLA_980 | MOCA | 0 | N.A | N.A | 1.65 [ -0.02; 3.32] | 1.65 [ -0.02; 3.32] | N.A |  |
| EVLA_980 | RFA | 0 | N.A | N.A | 1.05 [ -0.27; 2.37] | 1.05 [ -0.27; 2.37] | N.A |  |
| EVLA_980 | UGFS | 0 | N.A | N.A | 9.09 [ 7.13; 11.04] | 9.09 [ 7.13; 11.04] | N.A |  |
| MOCA | HL&S | 0 | N.A | N.A | -2.65 [ -4.03; -1.27] | -2.65 [ -4.03; -1.27] | N.A |  |
| RFA | HL&S | 3 | 89.30% | -2.04 [ -3.11; -0.97] | -2.07 [ -3.92; -0.22] | -2.05 [ -2.97; -1.12] | 0.9762 |  |
| UGFS | HL&S | 1 | N.A | -11.00 [-13.09; -8.91] | -8.20 [-11.21; -5.19] | -10.09 [-11.80; -8.37] | 0.1341 |  |
| MOCA | RFA | 2 | 40.70% | -0.60 [ -1.63; 0.42] | N.A | -0.60 [ -1.63; 0.42] | N.A |  |
| MOCA | UGFS | 0 | N.A | N.A | 7.44 [ 5.41; 9.46] | 7.44 [ 5.41; 9.46] | N.A |  |
| RFA | UGFS | 0 | N.A | N.A | 8.04 [ 6.29; 9.78] | 8.04 [ 6.29; 9.78] | N.A |  |
| **B. VCSS at 6 months** | | | | | | | | |
| **Arm 1** | **Arm 2** | **k** | **I^2^** | **Direct estimate** | **Indirect estimate** | **Network meta-analysis** | **Local Inconsistency** | **Global Inconsistency** |
| CAC | Electrocoagulation | 0 | N.A | N.A | 0.86 [ 0.39; 1.34] | 0.86 [ 0.39; 1.34] | N.A | *Q* = 1.46 *p* = 0.48 |
| CAC | EVLA_1470 | 2 | 38.80% | 0.04 [-0.10; 0.18] | -0.08 [-0.67; 0.51] | 0.03 [-0.11; 0.17] | 0.7075 |  |
| CAC | EVLA_980 | 0 | N.A | N.A | -0.22 [-0.53; 0.08] | -0.22 [-0.53; 0.08] | N.A |  |
| CAC | HL&S | 1 | N.A | 0.30 [ 0.02; 0.58] | 0.18 [-0.37; 0.74] | 0.28 [ 0.02; 0.53] | 0.7118 |  |
| CAC | MOCA | 0 | N.A | N.A | 0.44 [-0.58; 1.46] | 0.44 [-0.58; 1.46] | N.A |  |
| CAC | RFA | 2 | 0.00% | 0.00 [-0.39; 0.39] | 0.62 [-0.30; 1.55] | 0.09 [-0.26; 0.45] | 0.2244 |  |
| Electrocoagulation | EVLA_1470 | 0 | N.A | N.A | -0.83 [-1.32; -0.34] | -0.83 [-1.32; -0.34] | N.A |  |
| Electrocoagulation | EVLA_980 | 0 | N.A | N.A | -1.08 [-1.62; -0.55] | -1.08 [-1.62; -0.55] | N.A |  |
| Electrocoagulation | HL&S | 0 | N.A | N.A | -0.59 [-1.11; -0.06] | -0.59 [-1.11; -0.06] | N.A |  |
| Electrocoagulation | MOCA | 0 | N.A | N.A | -0.42 [-1.42; 0.58] | -0.42 [-1.42; 0.58] | N.A |  |
| Electrocoagulation | RFA | 1 | N.A | -0.77 [-1.08; -0.46] | N.A | -0.77 [-1.08; -0.46] | N.A |  |
| EVLA_1470 | EVLA_980 | 1 | N.A | -0.20 [-0.61; 0.21] | -0.32 [-0.77; 0.14] | -0.25 [-0.56; 0.05] | 0.7075 |  |
| EVLA_1470 | HL&S | 0 | N.A | N.A | 0.24 [-0.03; 0.52] | 0.24 [-0.03; 0.52] | N.A |  |
| EVLA_1470 | MOCA | 0 | N.A | N.A | 0.41 [-0.62; 1.44] | 0.41 [-0.62; 1.44] | N.A |  |
| EVLA_1470 | RFA | 0 | N.A | N.A | 0.06 [-0.32; 0.44] | 0.06 [-0.32; 0.44] | N.A |  |
| EVLA_980 | HL&S | 1 | N.A | 0.45 [ 0.06; 0.84] | 0.57 [ 0.08; 1.05] | 0.50 [ 0.19; 0.80] | 0.7118 |  |
| EVLA_980 | MOCA | 0 | N.A | N.A | 0.66 [-0.39; 1.71] | 0.66 [-0.39; 1.71] | N.A |  |
| EVLA_980 | RFA | 1 | N.A | 0.78 [-0.09; 1.65] | 0.16 [-0.35; 0.66] | 0.31 [-0.12; 0.75] | 0.2244 |  |
| MOCA | HL&S | 0 | N.A | N.A | -0.17 [-1.21; 0.88] | -0.17 [-1.21; 0.88] | N.A |  |
| RFA | HL&S | 0 | N.A | N.A | 0.18 [-0.24; 0.61] | 0.18 [-0.24; 0.61] | N.A |  |
| MOCA | RFA | 1 | N.A | -0.35 [-1.30; 0.60] | N.A | -0.35 [-1.30; 0.60] | N.A |  |


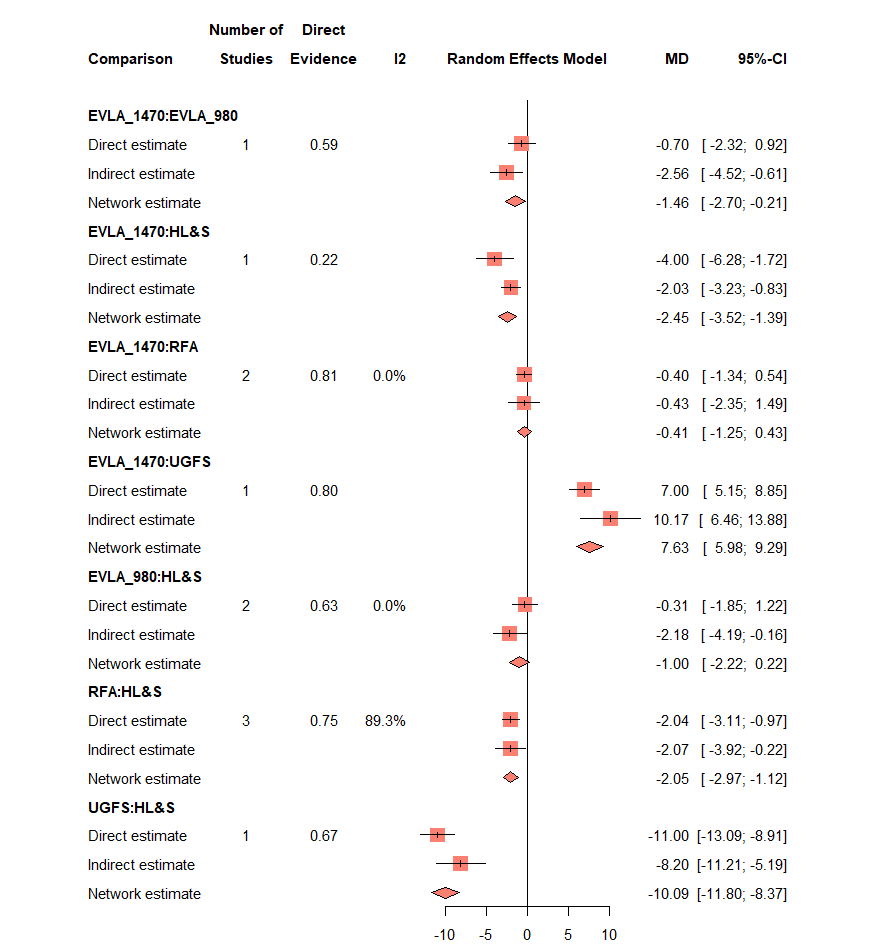


# **Figure S3**. Netsplit Forest Plot of Local Inconsistency Assessment for Time to Return to Normal Activities Network Meta-Analysis


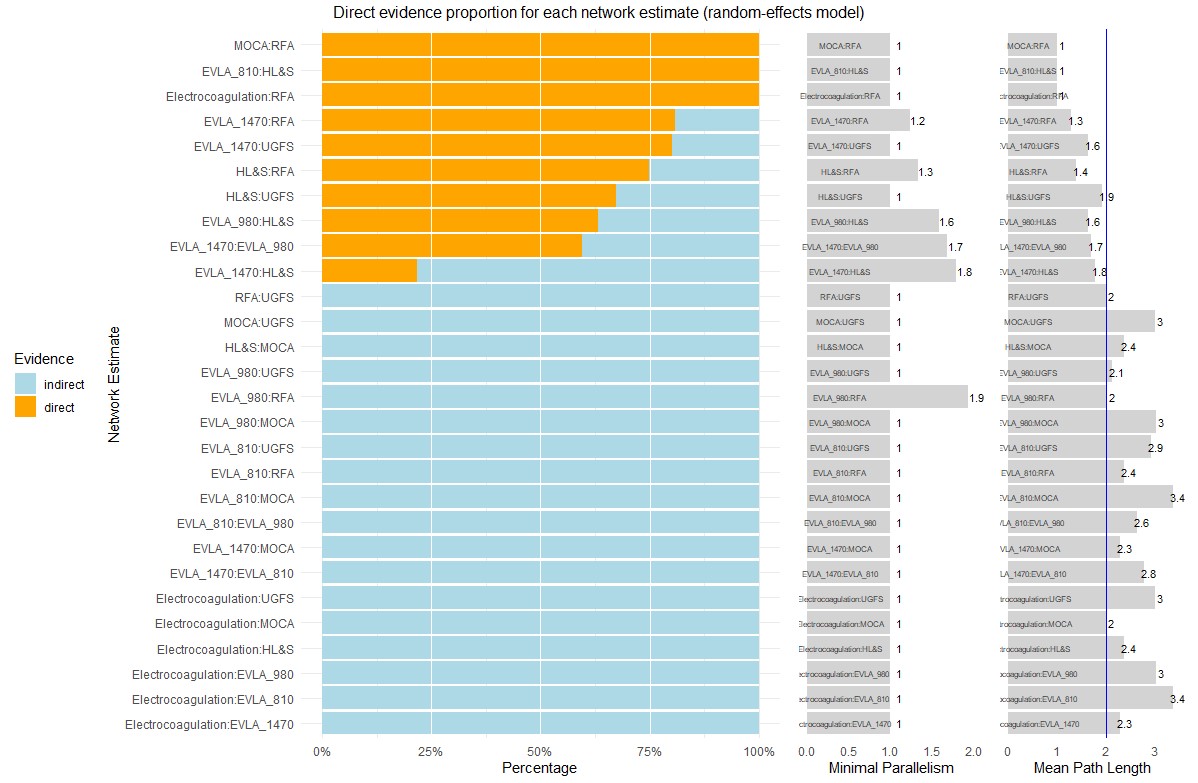


# **Figure S4.** Direct and Indirect Evidence Plot of Time to Return to Normal Activities Network Meta-Analysis


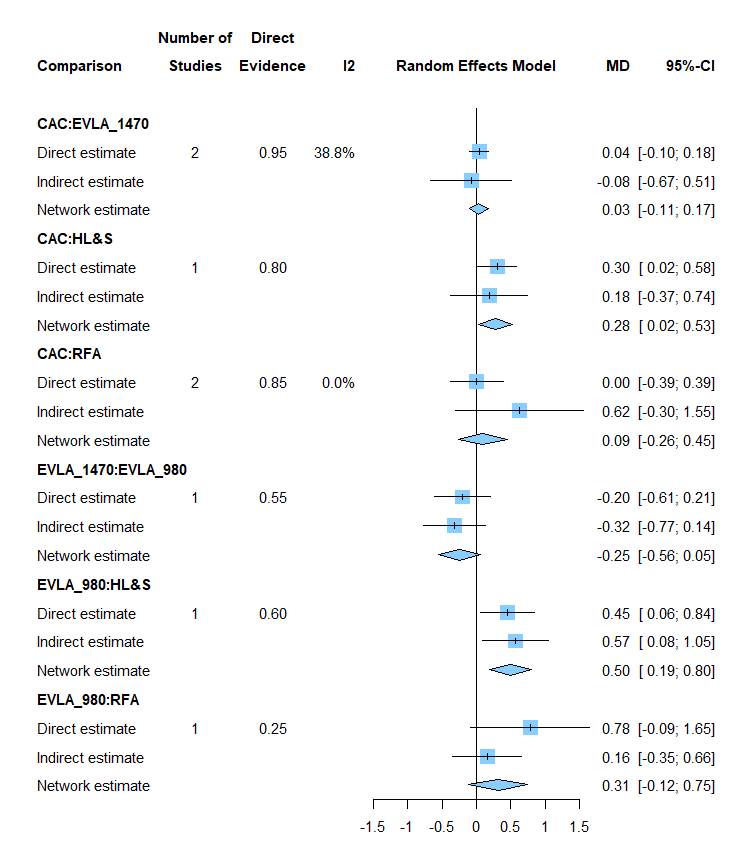


# **Figure S5**. Netsplit Forest Plot of Local Inconsistency Assessment for VCSS at 6 Months Network Meta-Analysis


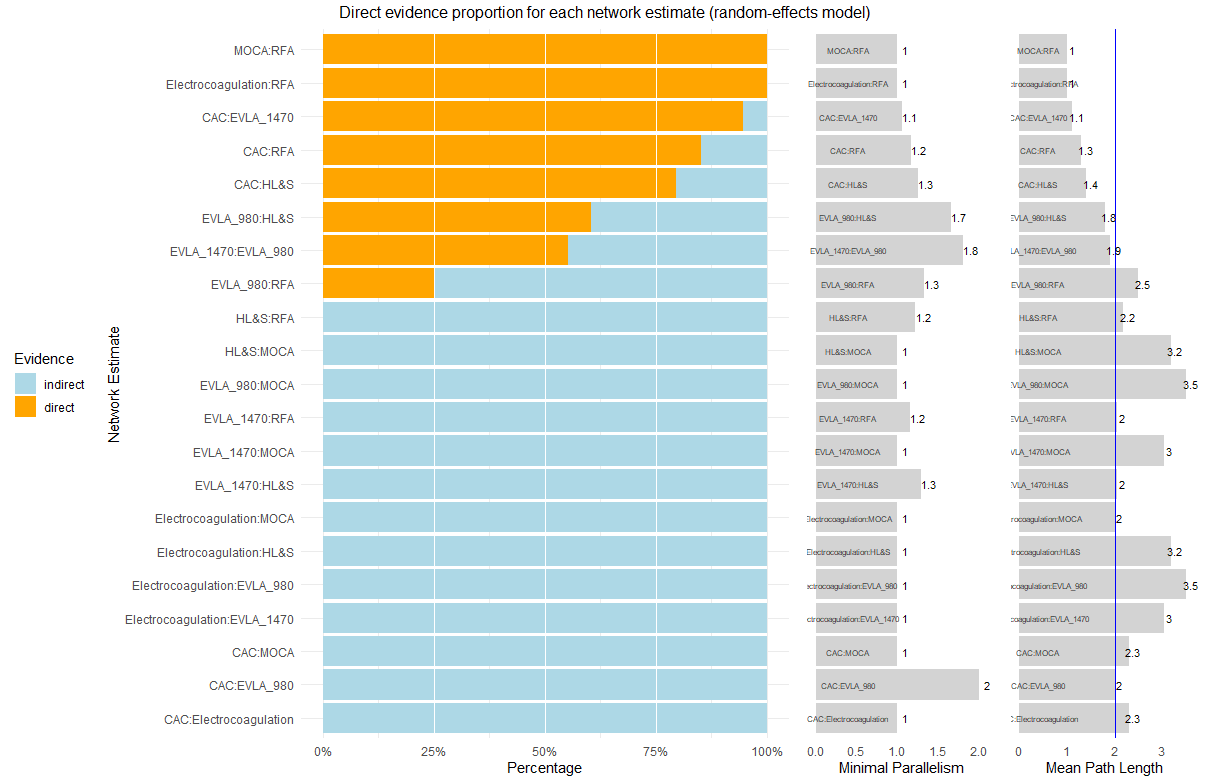


# **Figure S6.** Direct and Indirect Evidence Plot of VCSS at 6 Months Network Meta-Analysis


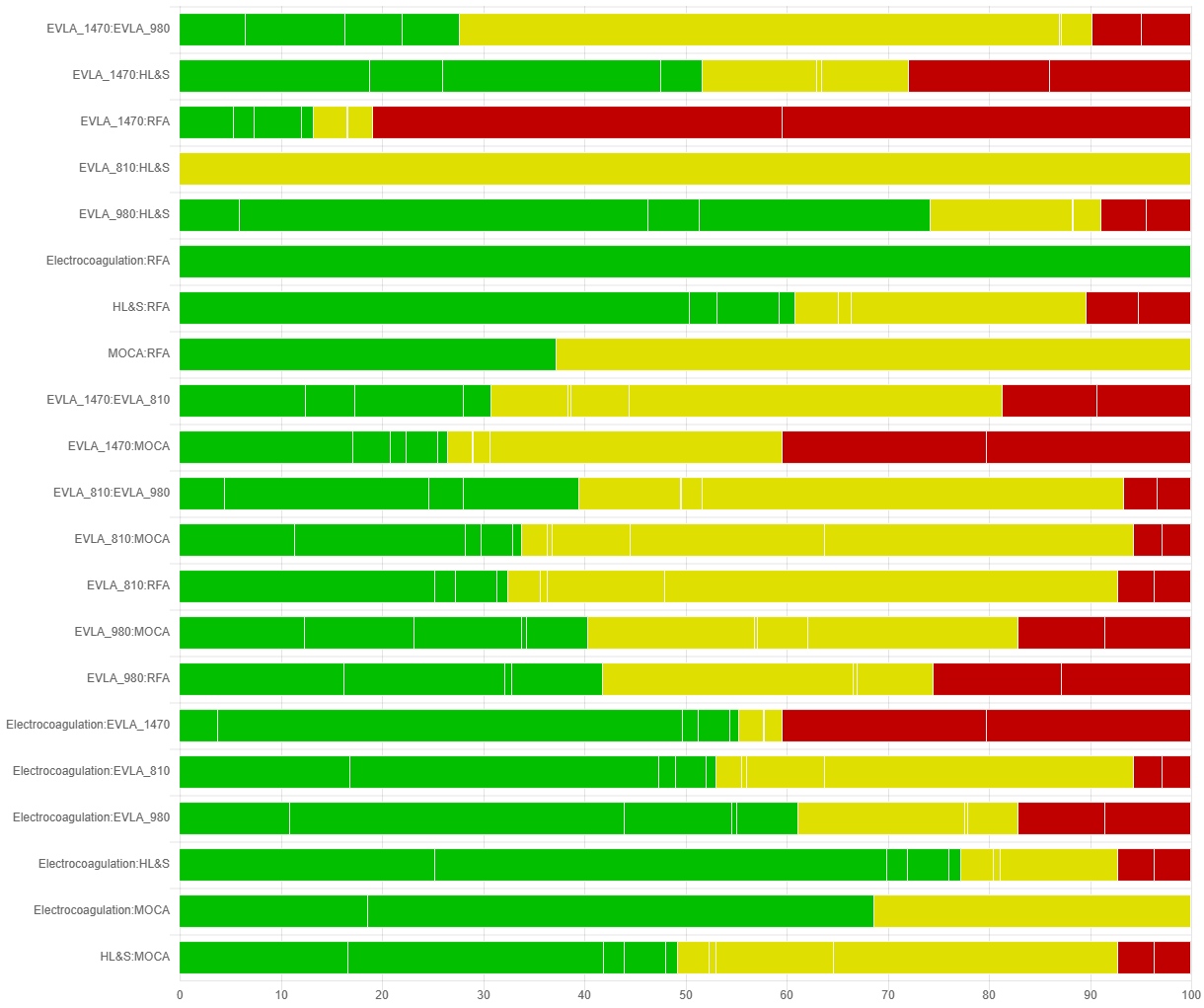


# **Figure S7**. Risk of Bias Contributions Within-Study Bias for Network Meta-Analysis of Time to Return to Normal Activities


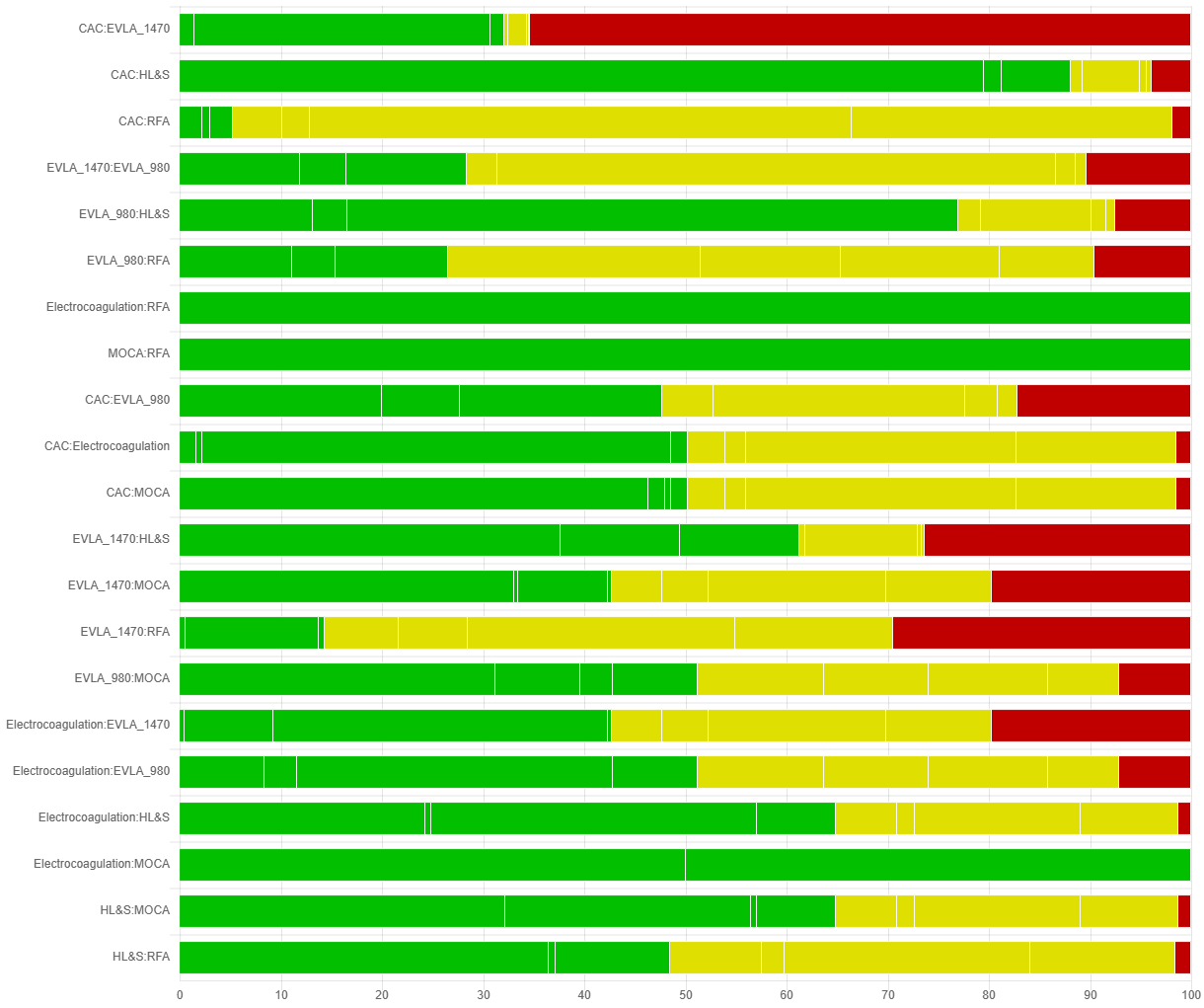


# **Figure S8**. Risk of Bias Contributions Within-Study Bias for Network Meta-Analysis of VCSS at 6 Months


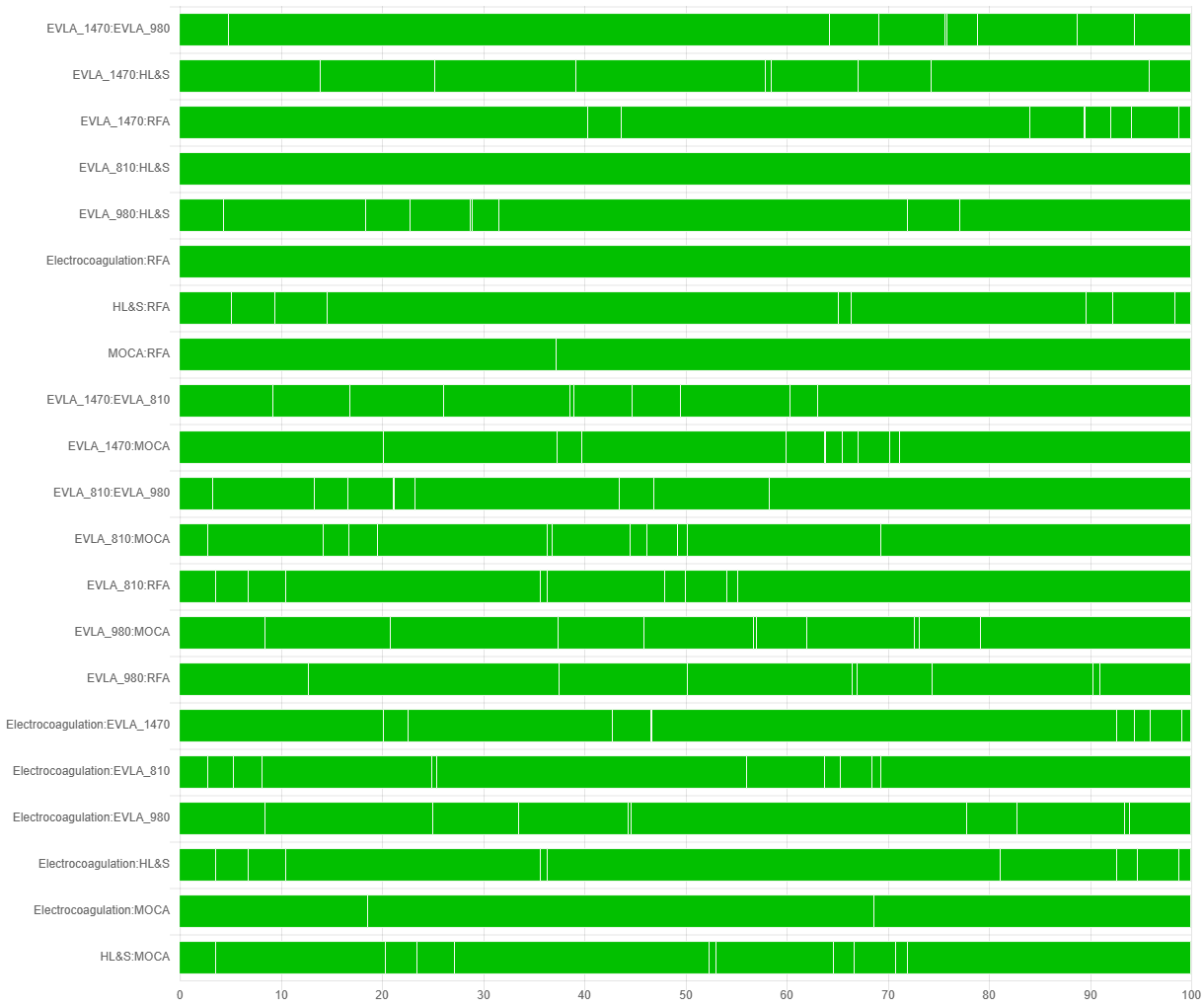


# **Figure S9**. Indirectness Contributions in GRADE Reports for Network Meta-Analysis of Time to Return to Normal Activities


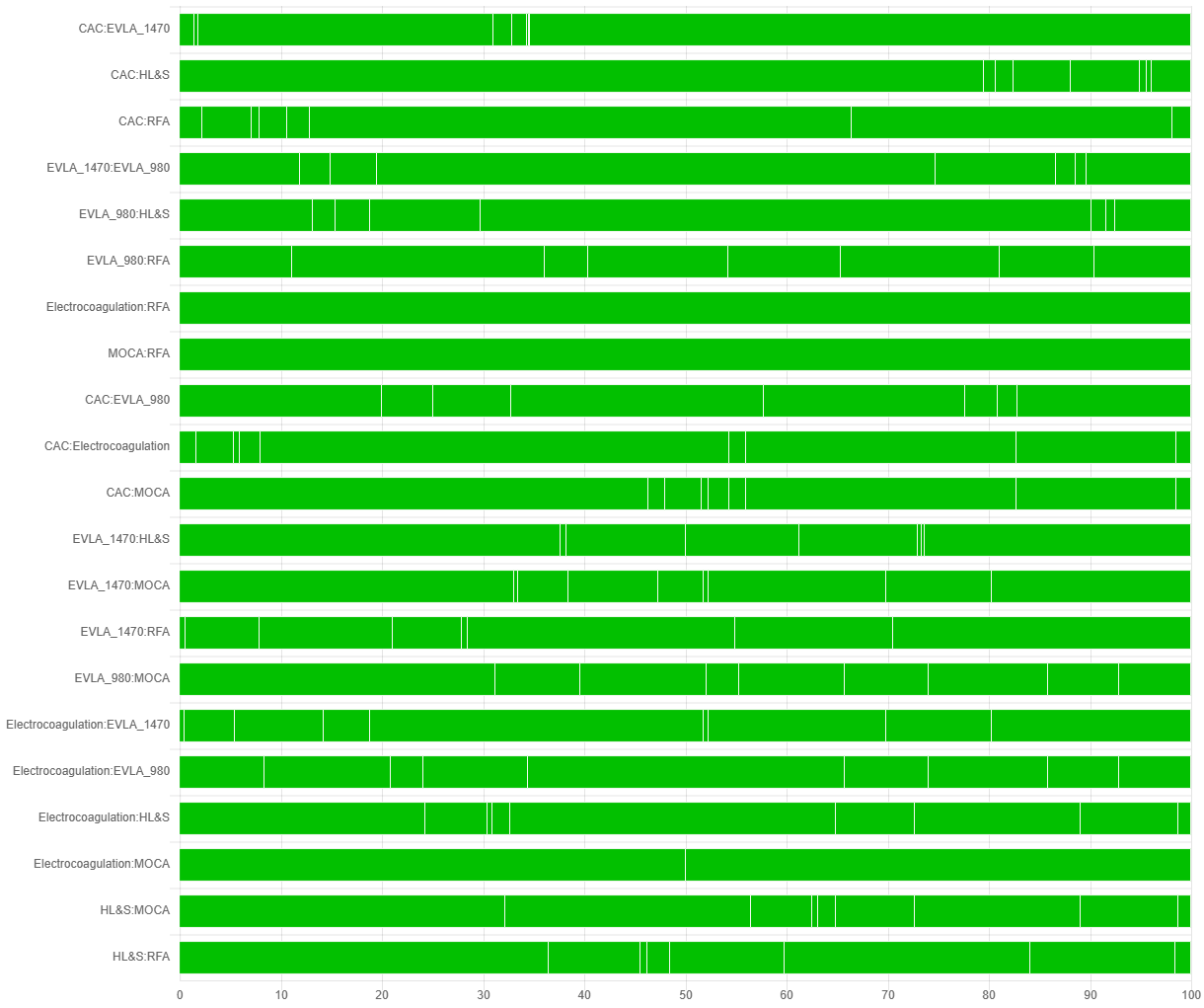


# **Figure S10**. Indirectness Contributions in GRADE Reports for Network Meta-Analysis of VCSS at 6 Months

# **Table S8.** GRADE Assessment of Network Estimates and Heterogeneity: Risk of Bias per Pairwise Treatment for Each Outcomes

| **A. Time to Return to Normal Activities** | | | | | | | | |
| --- | --- | --- | --- | --- | --- | --- | --- | --- |
| **Comparison** | **Number of Studies** | **Within Study Bias** | **Reporting bias** | **Indirectness** | **Imprecision** | **Heterogeneity** | **Incoherence** | **Confidence rating** |
| Mixed Evidence | | | | | | | | |
| EVLA_1470:EVLA_980 | 1 | Some concerns | Low risk | No concerns | No concerns | No concerns | No concerns | Moderate |
| EVLA_1470:HL&S | 1 | No concerns | Low risk | No concerns | No concerns | No concerns | No concerns | High |
| EVLA_1470:RFA | 2 | Major concerns | Low risk | No concerns | No concerns | No concerns | No concerns | Very low |
| EVLA_810:HL&S | 1 | Some concerns | Low risk | No concerns | No concerns | Some concerns | No concerns | Low |
| EVLA_980:HL&S | 2 | No concerns | Low risk | No concerns | No concerns | No concerns | No concerns | High |
| Electrocoagulation:RFA | 1 | No concerns | Low risk | No concerns | Some concerns | No concerns | No concerns | Moderate |
| HL&S:RFA | 3 | No concerns | Low risk | No concerns | No concerns | No concerns | No concerns | High |
| MOCA:RFA | 2 | Some concerns | Low risk | No concerns | No concerns | No concerns | No concerns | Moderate |
| EVLA_1470:EVLA_810 | 0 | Some concerns | Low risk | No concerns | Some concerns | No concerns | No concerns | Low |
| Electrocoagulation:EVLA_1470 | 0 | No concerns | Low risk | No concerns | Some concerns | No concerns | No concerns | Moderate |
| EVLA_1470:MOCA | 0 | Major concerns | Low risk | No concerns | No concerns | No concerns | No concerns | Very low |
| EVLA_810:EVLA_980 | 0 | Some concerns | Low risk | No concerns | No concerns | Some concerns | No concerns | Low |
| Electrocoagulation:EVLA_810 | 0 | No concerns | Low risk | No concerns | Some concerns | Some concerns | No concerns | Low |
| EVLA_810:MOCA | 0 | Some concerns | Low risk | No concerns | Some concerns | No concerns | No concerns | Low |
| EVLA_810:RFA | 0 | Some concerns | Low risk | No concerns | Some concerns | No concerns | No concerns | Low |
| Electrocoagulation:EVLA_980 | 0 | No concerns | Low risk | No concerns | No concerns | Some concerns | No concerns | Moderate |
| EVLA_980:MOCA | 0 | Some concerns | Low risk | No concerns | No concerns | No concerns | No concerns | Moderate |
| EVLA_980:RFA | 0 | No concerns | Low risk | No concerns | No concerns | No concerns | No concerns | High |
| Electrocoagulation:HL&S | 0 | No concerns | Low risk | No concerns | No concerns | No concerns | No concerns | High |
| Electrocoagulation:MOCA | 0 | No concerns | Low risk | No concerns | Some concerns | No concerns | No concerns | Moderate |
| HL&S:MOCA | 0 | No concerns | Low risk | No concerns | No concerns | No concerns | No concerns | High |
| **B. VCSS at 6 Months** | | | | | | | | |
| **Comparison** | **Number of Studies** | **Within Study Bias** | **Reporting bias** | **Indirectness** | **Imprecision** | **Heterogeneity** | **Incoherence** | **Confidence rating** |
| Mixed Evidence | | | | | | | | |
| CAC:EVLA_1470 | 2 | Major concerns | Low risk | No concerns | No concerns | No concerns | No concerns | Very low |
| CAC:HL&S | 1 | No concerns | Low risk | No concerns | No concerns | No concerns | No concerns | High |
| CAC:RFA | 2 | Some concerns | Low risk | No concerns | No concerns | No concerns | No concerns | Moderate |
| EVLA_1470:EVLA_980 | 1 | Some concerns | Low risk | No concerns | No concerns | No concerns | No concerns | Moderate |
| EVLA_980:HL&S | 1 | No concerns | Low risk | No concerns | No concerns | No concerns | No concerns | High |
| EVLA_980:RFA | 1 | Some concerns | Low risk | No concerns | No concerns | No concerns | No concerns | Moderate |
| Electrocoagulation:RFA | 1 | No concerns | Low risk | No concerns | No concerns | No concerns | No concerns | High |
| MOCA:RFA | 1 | No concerns | Low risk | No concerns | No concerns | No concerns | No concerns | High |
| CAC:EVLA_980 | 0 | No concerns | Low risk | No concerns | No concerns | No concerns | No concerns | High |
| CAC:Electrocoagulation | 0 | No concerns | Low risk | No concerns | No concerns | No concerns | No concerns | High |
| CAC:MOCA | 0 | No concerns | Low risk | No concerns | No concerns | No concerns | No concerns | High |
| Electrocoagulation:EVLA_1470 | 0 | No concerns | Low risk | No concerns | No concerns | No concerns | No concerns | High |
| EVLA_1470:HL&S | 0 | No concerns | Low risk | No concerns | No concerns | No concerns | No concerns | High |
| EVLA_1470:MOCA | 0 | No concerns | Low risk | No concerns | No concerns | No concerns | No concerns | High |
| EVLA_1470:RFA | 0 | Some concerns | Low risk | No concerns | No concerns | No concerns | No concerns | High |
| Electrocoagulation:EVLA_980 | 0 | No concerns | Low risk | No concerns | No concerns | No concerns | No concerns | High |
| EVLA_980:MOCA | 0 | No concerns | Low risk | No concerns | No concerns | No concerns | No concerns | High |
| Electrocoagulation:HL&S | 0 | No concerns | Low risk | No concerns | No concerns | No concerns | No concerns | High |
| Electrocoagulation:MOCA | 0 | No concerns | Low risk | No concerns | No concerns | No concerns | No concerns | High |
| HL&S:MOCA | 0 | No concerns | Low risk | No concerns | No concerns | No concerns | No concerns | High |
| HL&S:RFA | 0 | Some concerns | Low risk | No concerns | No concerns | No concerns | No concerns | High |
| Judgments for the six domains across all evaluated treatment effects are reported, except inconherence. The default summary judgment is “High” confidence; downgrading by one, two, or three levels will lead to a confidence rating of “Moderate,” “Low,” or “Very low” respectively. We manually assign an overall level of confidence to each relative effect. **GRADE**, Grading of Recommendations Assessment, Development and Evaluation. | | | | | | | | |
